# Complexity and Expressive Power of Weakly Well-Designed SPARQL

**DOI:** 10.1007/s00224-017-9802-9

**Published:** 2017-08-14

**Authors:** Mark Kaminski, Egor V. Kostylev

**Affiliations:** Department of Computer Science, University of Oxford, Oxford, UK

**Keywords:** RDF query languages, SPARQL, Optional matching

## Abstract

SPARQL is the standard query language for RDF data. The distinctive feature of SPARQL is the OPTIONAL operator, which allows for partial answers when complete answers are not available due to lack of information. However, optional matching is computationally expensive—query answering is PSPACE-complete. The well-designed fragment of SPARQL achieves much better computational properties by restricting the use of optional matching—query answering becomes coNP-complete. On the downside, well-designed SPARQL captures far from all real-life queries—in fact, only about half of the queries over DBpedia that use OPTIONAL are well-designed. In the present paper, we study queries outside of well-designed SPARQL. We introduce the class of weakly well-designed queries that subsumes well-designed queries and includes most common meaningful non-well-designed queries: our analysis shows that the new fragment captures over 99% of DBpedia queries with OPTIONAL. At the same time, query answering for weakly well-designed SPARQL remains coNP-complete, and our fragment is in a certain sense maximal for this complexity. We show that the fragment’s expressive power is strictly in-between well-designed and full SPARQL. Finally, we provide an intuitive normal form for weakly well-designed queries and study the complexity of containment and equivalence.

## Introduction

The Resource Description Framework (RDF) [14, 18, 31] is the W3C standard for representing linked data on the Web. RDF models information in terms of labelled graphs consisting of triples of resource identifiers (IRIs). The first and last IRIs in such a triple, called *subject* and *object*, represent entity resources, while the middle IRI, called *predicate*, represents a relation between the two entities.

SPARQL [17, 37] is the default query language for RDF graphs. First standardised in 2008 [37], SPARQL is now recognised as a key technology for the Semantic Web. This is witnessed by a recent adoption of a new version of the standard, SPARQL 1.1 [17], as well as by active development of SPARQL query engines in academia and industry, for instance, as part of the systems AllegroGraph (http://franz.com/agraph/allegrograph/), Apache Jena (http://jena.apache.org), RDF4J (http://rdf4j.org), or OpenLink Virtuoso (http://virtuoso.openlinksw.com).

In recent years, SPARQL has been subject to a substantial amount of theoretical research, based on the foundational work by Pérez et al. [32, 33]. In particular, we now know much about evaluation [1, 3, 4, 6, 8, 20, 22, 23, 25, 28, 34, 38], optimisation [8, 9, 12, 13, 24, 27, 35], federation [10, 11], expressive power [2, 20, 21, 25, 36, 39], and provenance tracking [15, 16] for queries from various fragments and extensions of SPARQL. These studies have had a great impact in the community, in fact influencing the evolution of SPARQL as a standard.

**Fig. 1:**
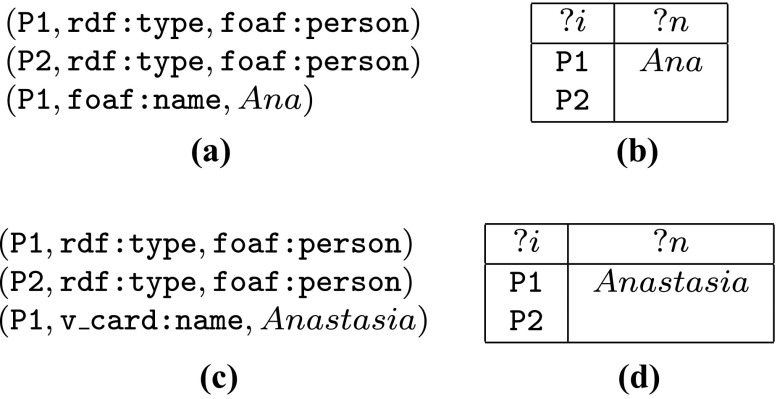
**a** Graph *G*; **b** answers to query (1) over *G*; **c** graph *G* ^′^; and **d** answers to (2) over *G* ^′^

A distinctive feature of SPARQL as compared to SQL is the O P T I O N A L operator (abbreviated as O P T in this paper). This operator was introduced to “*not reject (solutions) because some part of the query pattern does not match*” [37]. For instance, consider the SPARQL query

1 $$ \begin{array}{l} \mathsf{SELECT} \; ?i, ?n\\ \qquad \; \mathsf{WHERE} \; {(?i, \texttt{rdf:type}, \texttt{foaf:person})} ~{{\mathsf{OPT}}}~{(?i, \texttt{foaf:name}, ?n)}, \end{array} $$

which retrieves all person IDs from the graph together with their names; names, however, are optional—if the graph does not contain information about the name of a person, the person ID is still retrieved but the variable ?*n* is left undefined in the answer. For instance, query (1) has two answers over the graph *G* in Fig. 1a, where the second answer is partial (see Fig. 1b). However, if we extend *G* with a triple supplying a name for P2, the second answer will include this name.

The O P T operator accounts in a natural way for the open world assumption and the fundamental incompleteness of the Web. However, evaluating queries that use O P T is computationally expensive—Pérez et al. [33] showed PSpace-completeness of SPARQL query evaluation, and Schmidt et al. [38] refined this result by proving PSpace-hardness even for queries using no operators besides O P T. This is not surprising given that SPARQL queries are equivalent in expressive power to first-order logic queries, and translations in both directions can be done in polynomial time [2, 25, 36].

This spurred a search for restrictions on the use of O P T that would ensure lower complexity of query evaluation. It was also recognised that queries that are difficult to evaluate are often unintuitive. For instance, they may produce less specified answers (i.e., answers with fewer bound variables) as the graph over which they are evaluated grows larger.

Pérez et al. [33] introduced the *well-designed* fragment of SPARQL queries by imposing a syntactic restriction on the use of variables in O P T-expressions. Roughly speaking, each variable in the optional (i.e., right) argument of an O P T-expression should either appear in the mandatory (i.e., left) argument or be globally fresh for the query, i.e., appear nowhere outside of the argument. Well-designed queries have lower complexity of query evaluation—the problem is coNP-complete (provided all the variables in the query are selected). Moreover, such queries have a more intuitive behaviour than arbitrary SPARQL queries; in particular, they enjoy the monotonicity property that we observed for query (1): each partial answer over a graph can potentially be extended to undefined variables if the graph is completed with the missing information, and the more information we have the more specified are the answers. Well-designed queries can be efficiently transformed to an intuitive normal form allowing for a transparent graphical representation of queries as trees [27, 35]. Hence, many recent studies concentrate partially [23, 25, 27, 40, 41] or entirely [1, 8, 35] on well-designed queries.

Such a success of well-designed queries may lead to the impression that non-well-designed SPARQL queries are just a useless side effect of the early specification. But is this impression justified by the use of SPARQL in practice? To answer this question, a comprehensive analysis of real-life queries is required. We are aware of two works that analyse the distribution of operators in SPARQL queries asked over DBpedia [7, 34]. Both studies show that O P T is used in a non-negligible amount of practical queries. However, only Picalausa and Vansummeren [34] go further and analyse how many of these queries are well-designed; and the result is quite interesting—well-designed queries make up only about half of all queries with O P T. In other words, well-designed queries are common, but by far not exclusive.

The main goal of this paper is to investigate SPARQL queries beyond the well-designed fragment. We wanted to see if the well-designedness condition could be extended so as to include most practical queries while preserving good computational properties. The main result of our study is very positive—we identified a new fragment of SPARQL queries, called *weakly well-designed* queries, that covers over 99% of queries over DBpedia and has the same complexity of query evaluation as the well-designed fragment. We also show that our fragment is in a sense maximal for this complexity.

We next describe our results and techniques in more detail. Our first step was to identify typical real-life queries that are not well-designed. We analysed DBpedia query logs in recent USEWOD research datasets [29, 30] and found two interesting types of non-well-designed queries. The first type is exemplified by the following query:

2 $$ \begin{array}{l} \mathsf{SELECT} \; ?i, ?n \\ \qquad \; \mathsf{WHERE} \; ({(?i, \texttt{rdf:type}, \texttt{foaf:person})}~~{{\mathsf{OPT}}}~~{(?i, \texttt{foaf:name}, ?n)}) \\ \qquad \qquad \qquad \qquad \qquad \qquad \qquad \qquad \qquad \qquad ~~~{{\mathsf{OPT}}}~ ~{(?i, \texttt{v\_card:name}, ?n)}. \end{array} $$

This query is clearly not well-designed because variable ?*n*, binding the name of a person, appears in two different unrelated optional parts. Let us analyse answers to this query over different graphs. On graph *G* in Fig. 1a the result is exactly the same as for query (1), shown in Fig. 1b, simply because the IRI v_card:name is not present in *G*, and so cannot be matched against the second optional part of the query. Similarly, on graph *G* ^′^ in Fig. 1c, where the source of the name and the name itself are different, the result is as in Fig. 1d. In this case, the first optional part in the query does not match anything in the graph so the variable ?*n* is left unbound at this point; then the second optional is matched, and the variable is assigned with the name from v_card. More interestingly, query (2) evaluated over the graph *G* ∪ *G* ^′^ once again yields the result in Fig. 1b. Indeed, in this case, the first optional part has a match again and ?*n* is assigned the value *A* *n* *a*; then, this variable is already bound and there is no match for the second optional part that agrees with this value, meaning that the alternative v_card name is disregarded by the query. To summarise, query (2) is once again looking for person IDs and, optionally, their names. Now, however, names are collected from two different sources, foaf and v_card, where the first source is given preference over the second (maybe because it is considered more reliable or more informative, or for some other reason). In other words, if we know the foaf name of a person, it is returned as part of the answer regardless of their v_card name; however, if there is no foaf name, then the v_card name is also acceptable and should be returned; variable ?*n* is left unbound only if the name cannot be extracted from either source.

Of course, preference patterns encountered in real-life queries are often more complex. Still, we will see that in most cases they do not increase the complexity of query evaluation.

Our second example query is as follows:

3 $$ \begin{array}{l} \mathsf{SELECT} \; ?i, ?n \\ \qquad \; \mathsf{WHERE} \; ({(?i, \texttt{rdf:type}, \texttt{foaf:person})}~{{\mathsf{OPT}}}~{(?i, \texttt{foaf:name}, ?n)}) \\ \qquad \qquad \qquad \qquad \qquad \qquad \qquad \; {} \mathsf{FILTER}~ (\neg bound (?n) \vee \neg (?n = Ana)). \end{array} $$

The query uses F I L T E R, a standard SPARQL operator that admits only answers conforming to a specified constraint. Again, this query is not well-designed because the F I L T E R constraint mentions the variable ?*n*, which occurs in the optional part of the query but not in the mandatory part. However, the intention of the query is quite clear: it searches for people whose names are not known to be *A* *n* *a*, including people whose names are unknown.

This use of F I L T E R is in fact very common in real-life queries. Moreover, it is intuitive as long as F I L T E R is essentially the outermost operator in the query, as it is in our example. We will see that in all such cases F I L T E R cannot lead to an increase in complexity.

Having isolated these typical uses of non-well-de-signed-ness, we identify a new fragment of SPARQL that (a) includes all queries of the above two types, (b) subsumes well-designed queries, and (c) has the same complexity of query evaluation as well-designed queries. We call such queries *weakly well-designed*. They are the maximal fragment without structural restrictions on conjunctive blocks and filter conditions that has the above properties. Our analysis shows that more than 99% of DBpedia queries with O P T are weakly well-designed.

Besides low complexity of query evaluation, we establish a few more useful properties of weakly well-designed queries, which are summarised in the following outline of the paper. After introducing the syntax and semantics of SPARQL in Section 2, we formally define our new fragment in Section 3. In Section 4, we show that, similarly to the well-designed case, weakly well-designed queries can be transformed to an intuitive normal form, which allows for a natural graphical representation as *constraint pattern trees*. Using this representation, in Section 5, we formally show that the step from well-designed to weakly well-designed queries does not increase complexity of query evaluation; minimal relaxations of weak well-designedness, however, already lead to a complexity jump. In Section 6, we compare the expressive power of our fragment (and its extensions with additional operators) with well-designed queries and unrestricted SPARQL queries; in all cases, we show that the expressivity of weakly well-designed queries lies strictly in-between well-designed and unrestricted queries. In Section 7, we study static analysis problems for weakly well-designed queries and establish ${{\Pi }_{2}^{p}}$-completeness of equivalence and containment. Finally, in Section 8, we detail our analysis of DBpedia logs.

This article significantly extends the conference paper [19]. Besides providing full proofs of our technical claims, we have extended the analysis section and updated the evaluation to use more recent datasets. Furthermore, we have removed the erroneous claim that queries over unions of weakly well-designed patterns have the same expressive power as unrestricted SPARQL queries; on the contrary, we show that the former are strictly less expressive than the latter.

## SPARQL Query Language

We begin by formally introducing the syntax and semantics of SPARQL that we adopt in this paper. Our formal setup mostly follows [33], which has some differences from the W3C specification [17, 37]; in particular, we use two-placed O P T and two-valued F I L T E R (conditional O P T and errors in F I L T E R evaluation as in the standard are expressible in our formalisation [2, 21]), do not consider blank nodes (their presence in RDF graphs would not change any of our results), and adopt set semantics, leaving multiset answers for future work.

### **RDF Graphs**

An RDF graph is a labelled graph where nodes can also serve as edge labels. Formally, let **I** be a set of *IRIs*. Then an *RDF triple* is a tuple (*s*, *p*, *o*) from **I** × **I** × **I**, where *s* is called *subject*, *p* *predicate*, and *o* *object*. An *RDF graph* is a finite set of RDF triples.

### **SPARQL Syntax**

Let **X** be an infinite set {?*x*, ?*y*, …} of *variables*, disjoint from **I**. *Filter constraints* are conditions of the form

- ⊤, ?*x* = *u*, ?*x* = ?*y*, or *b* *o* *u* *n* *d*(?*x*) for ?*x*, ?*y* in **X** and *u* ∈ **I** (these constraints are called *atomic*),
- ¬*R*_1_, *R*_1_ ∧ *R*_2_, or *R*_1_ ∨ *R*_2_ for filter constraints *R*_1_ and *R*_2_.

A *basic pattern* is a possibly empty set of triples from (**I** ∪ **X**) × (**I** ∪ **X**) × (**I** ∪ **X**) (to avoid notational clutter, in examples we will often omit braces when writing singleton basic patterns, e.g., we will write (?*x*, *u*, ?*y*) instead of {(?*x*, *u*, ?*y*)}). Then, SPARQL *(graph) patterns* *P* are defined by the grammar

$$P \; ::= \; B \mid (P~{{\mathsf{AND}}}~P) \mid (P~ {{\mathsf{OPT}}}~ P) \mid (P~ {{\mathsf{UNION}}}~ P) \mid (P ~{{\mathsf{FILTER}}}~ R), $$

where *B* ranges over basic patterns and *R* over filter constraints. Additionally, we require all filter constraints to be *safe*, that is, *v* *a* *r* *s*(*R*) ⊆ *v* *a* *r* *s*(*P*) for every pattern (*P* F I L T E R *R*), where *v* *a* *r* *s*(*S*) is the set of all variables in *S* (which can be a pattern, constraint, etc.) When needed, we distinguish between patterns by their top-level operator; e.g., we write O P T-pattern or F I L T E R-pattern.

We write $\mathcal {U}$ for the set of all patterns. We also distinguish the fragment $\mathcal {P}$ of $\mathcal {U}$ that consists of all U N I O N-*free* patterns, that is, patterns that do not use the U N I O N operator.

Projection is realised in SPARQL by means of *queries with select result form*, or *queries* for short, which are expressions of the form

4 $$ \mathsf{SELECT} \; X \; \mathsf{WHERE} \; P, $$

where *X* is a set of variables and *P* is a graph pattern. We write $\mathcal {S}$ for the set of all queries. The set of all triples in basic patterns of a query *Q* is denoted t r i p l e s(*Q*).

Note that every pattern *P* can be seen as a query of the form (4) where *X* = *v* *a* *r* *s*(*P*). Hence, all definitions that refer to “queries” implicitly extend to patterns in the obvious way.

### **SPARQL Semantics**

The semantics of graph patterns is defined in terms of *mappings*, that is, partial functions from variables to IRIs. The *domain* *d* *o* *m*(*μ*) of a mapping *μ* is the set of variables on which *μ* is defined. Two mappings *μ*_1_ and *μ*_2_ are *compatible* (written *μ*_1_ ∼ *μ*_2_) if *μ*_1_(?*x*) = *μ*_2_(?*x*) for all variables ?*x* ∈ *d* *o* *m*(*μ*_1_) ∩ *d* *o* *m*(*μ*_2_). If *μ*_1_ ∼ *μ*_2_, then *μ*_1_ ∪ *μ*_2_ constitutes a mapping with domain *d* *o* *m*(*μ*_1_) ∪ *d* *o* *m*(*μ*_2_) that coincides with *μ*_1_ on *d* *o* *m*(*μ*_1_) and with *μ*_2_ on *d* *o* *m*(*μ*_2_). Given two sets of mappings Ω_1_ and Ω_2_, we define their *join*, *union* and *difference* as follows:

$$\begin{array}{ccl} {\Omega}_{1} \Join {\Omega}_{2} & = & \{\mu_{1} \cup \mu_{2} \mid \mu_{1} \in {\Omega}_{1}, \mu_{2} \in {\Omega}_{2}, \text{ and } \mu_{1} \sim \mu_{2}\}, \\ {\Omega}_{1} \cup {\Omega}_{2} & = & \{\mu \mid \mu \in {\Omega}_{1} \text{ or } \mu \in {\Omega}_{2}\}, \\ {\Omega}_{1} \setminus {\Omega}_{2} & = & \{\mu_{1} \mid \mu_{1} \in {\Omega}_{1}, \mu_{1} \not\sim \mu_{2} \text{ for all }\mu_{2} \in {\Omega}_{2}\}. \end{array} $$

Based on these, the *left outer join* operation is defined as 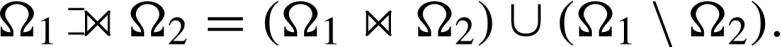


Given a graph *G*, the *evaluation* [[*P*]]_*G*_ of a graph pattern *P* over *G* is defined as follows:

1. if *B* is a basic pattern, then [[*B*]]_*G*_ = {*μ* : *v* *a* *r* *s*(*B*) → **I**∣*μ*(*B*) ⊆ *G*};
2. [[(*P*_1_ A N D *P*_2_)]]_*G*_ = [[*P*_1_]]_*G*_ ⋈ [[*P*_2_]]_*G*_;

4. [[(*P*_1_ U N I O N *P*_2_)]]_*G*_ = [[*P*_1_]]_*G*_ ∪ [[*P*_2_]]_*G*_;
5. [[(*P* ^′^ F I L T E R *R*)]]_*G*_ = {*μ*∣*μ* ∈ [[*P* ^′^]]_*G*_ and *μ* ⊧ *R*}, where *μ* *satisfies* a filter constraint *R*, denoted by *μ* ⊧ *R*, if one of the following holds:
*R* is ⊤;
*R* is ?*x* = *u*, ?*x* ∈ *d* *o* *m*(*μ*), and *μ*(?*x*) = *u*;
*R* is ?*x* = ?*y*, {?*x*, ?*y*}⊆ *d* *o* *m*(*μ*), and *μ*(?*x*) = *μ*(?*y*);
*R* is *b* *o* *u* *n* *d*(?*x*) and ?*x* ∈ *d* *o* *m*(*μ*);
*R* is a Boolean combination of filter constraints evaluating to *true* under the usual interpretation of ¬, ∧, and ∨.

Let *μ*|_*X*_ be the *projection* of a mapping *μ* to variables *X*, that is, *μ*|_*X*_(?*x*) = *μ*(?*x*) if ?*x* ∈ *X* and *μ*|_*X*_(?*x*) is undefined if ?*x* ∉ *X*. The *evaluation* [[*Q*]]_*G*_ of a query *Q* of the form (4) is the set of all mappings *μ*|_*X*_ such that *μ* ∈ [[*P*]]_*G*_.

Finally, a *solution* to a query (or pattern) *Q* over *G* is a mapping *μ* such that *μ* ∈ [[*Q*]]_*G*_.

## Weakly Well-Designed Patterns

We begin by recalling the notion of well-designed patterns and then formulate our generalisation. For now, we focus on the fragment $\mathcal {P}$ of U N I O N-free patterns (also known as the A N D- O P T- F I L T E R fragment of SPARQL), leaving the operators U N I O N and S E L E C T for later sections.

Note that a given pattern can occur more than once within a larger pattern. In what follows we will sometimes need to distinguish between a (sub-)pattern *P* as a possibly repeated building block of another pattern *P* ^′^ and its *occurrences* in *P* ^′^, that is, unique subtrees in the parse tree. Then, the *left (right) argument* of an occurrence *i* is the subtree rooted in the left (right) child of the root of *i* in the parse tree, and an occurrence *i* is *inside* an occurrence *j* if the root of *i* is a descendant of the root of *j*.

### **Definition 1**

(Pérez et al. [33]) A pattern *P* from $\mathcal {P}$ is *well-designed* (or *wd-pattern*, for short) if for every occurrence *i* of an O P T-pattern *P*_1_ O P T *P*_2_ in *P* the variables from *v* *a* *r* *s*(*P*_2_) ∖ *v* *a* *r* *s*(*P*_1_) occur in *P* only inside (the labels of) *i*.

We write $\mathcal {P}_{\text {wd}}$ for the fragment of wd-patterns. Such patterns comply with the basic intuition for optional matching in SPARQL: “*do not reject (solutions) because some part of the query pattern does not match*” [37]; indeed, our canonical use case (1) is clearly well-designed. Evaluation of wd-patterns, that is, checking if *μ* ∈ [[*P*]]_*G*_ for a mapping *μ*, graph *G* and pattern $P\in \mathcal {P}_{\text {wd}}$, is coNP-complete (in combined complexity), as opposed to PSpace-completeness for $\mathcal {P}$ [33, 38]. The high complexity of unrestricted patterns is partially due to the fact that unrestricted combinations of O P T and F I L T E R allow to express nesting of the difference operator D I F F with semantics [[*P*_1_ D I F F *P*_2_]]_*G*_ = [[*P*_1_]]_*G*_∖[[*P*_2_]]_*G*_ (unless *P*_1_ or *P*_2_ are empty basic patterns, see [21] for details):

5 $$ \begin{array}{l} P_{1}~ {\mathsf{DIFF}}~ P_{2} \equiv (P_{1}~ {{\mathsf{OPT}}}~(P_{2}~ {{\mathsf{AND}}}~ (?x, ?y, ?z))) ~{{\mathsf{FILTER}}}~ \neg bound(?x), \end{array} $$

where ?*x*, ?*y* and ?*z* do not occur in *v* *a* *r* *s*(*P*_1_) ∪ *v* *a* *r* *s*(*P*_2_). This property is well-known [2, 21, 33], and has been usually believed to be an important source of non-well-designed patterns in practice. We challenge this belief by answering differently the question on the prevalent structure of real-life queries beyond the well-designed fragment. This question is not just of theoretical interest: as previous studies [34] show (and our analysis confirms), about half of queries with O P T asked over DBpedia are not well-designed.

Next we discuss two sources of non-well-designedness in patterns as revealed by the example queries (2) and (3) in the introduction—one based on O P T and another one on F I L T E R.

*Source 1.*: There are two substantially different ways of nesting the O P T operator in patterns:
  Opt-R $$\begin{array}{@{}rcl@{}} P_{1}~ {{\mathsf{OPT}}}~ (P_{2}~ {{\mathsf{OPT}}}~ P_{3}), \end{array} $$
  Opt-L $$\begin{array}{@{}rcl@{}} (P_{1} ~{{\mathsf{OPT}}}~ P_{2})~{{\mathsf{OPT}}}~P_{3}. \end{array} $$
  Non-well-designed nesting of type (Opt-R) is responsible for the PSpace-hardness of query evaluation [33, 38]. Moreover, such nesting is not very intuitive unless well-designed. On the contrary, as we saw in the introduction, non-well-designed nesting of type (Opt-L) can be used for prioritising some parts of patterns to others, and is indeed used in real life. As we will see later, nesting of type (Opt-L) cannot lead to high complexity of evaluation.
*Source 2.*: Well-designedness can be violated by using “dangerous” variables from the right argument of O P T in filter constraints. In particular, patterns of the form (*P*_1_ O P T *P*_2_) F I L T E R *R* with *R* using a variable from *v* *a* *r* *s*(*P*_2_) ∖*v* *a* *r* *s*(*P*_1_) are not well-designed, but rather frequent in practice. However, such patterns almost never occur inside the right argument of other O P T-patterns. We will see that if we restrict the usage of such filters to the “top level”, we preserve the good computational properties of wd-patterns.

Motivated by these observations, we considerably generalise the notion of wd-patterns to allow for useful queries like (2) and (3) while retaining important properties of such patterns. We start with two auxiliary notions.

### **Definition 2**

Given a pattern *P*, an occurrence *i*_1_ in *P* dominates an occurrence *i*_2_ if there exists an occurrence *j* of an O P T-pattern such that *i*_1_ is inside the left argument of *j* and *i*_2_ is inside the right argument.

### **Definition 3**

An occurrence *i* of a F I L T E R-pattern *P* ^′^ F I L T E R *R* in *P* is *top-level* if there is no occurrence *j* of an O P T-pattern such that *i* is inside the right argument of *j*.

We are ready to give the main definition of this paper.

### **Definition 4**

A pattern $P \in \mathcal {P}$ is *weakly well-designed* (or *wwd-pattern*, for short) if, for each occurrence *i* of an O P T-subpattern *P*_1_ O P T *P*_2_, the variables in *v* *a* *r* *s*(*P*_2_)∖*v* *a* *r* *s*(*P*_1_) appear outside *i* only in

- subpatterns whose occurrences are dominated by *i*, and
- constraints of top-level occurrences of F I L T E R-patterns.

We write $\mathcal {P}_{\text {wwd}}$ for the fragment of wwd-patterns. They extend wd-patterns by allowing variables from the right argument of an O P T-subpattern that are not “guarded” by the left argument to appear in certain positions outside of the subpattern. Note that the patterns of queries (2) and (3) are wwd-patterns. Also, patterns which allow only for O P T nesting of type (Opt-L) are always weakly well-designed, same as the pattern on the right hand side of (5), which expresses D I F F. However, patterns that have subpatterns of the atter form in the right argument of O P T are not weakly well-designed. Next we give a few more examples.

### *Example 1*

**Fig. 2:**
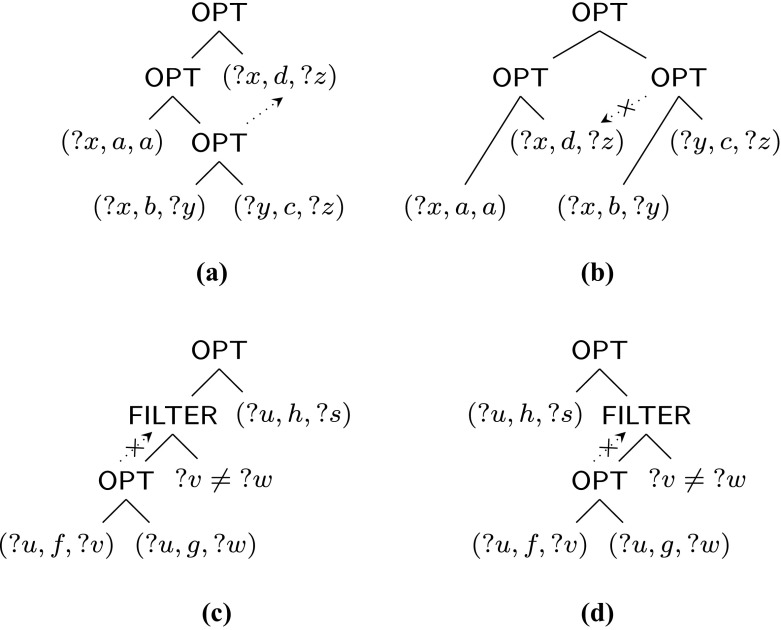
**a**–**d** Parse trees for patterns (6)–(9) in Example 1, respectively; (*crossed*) *dotted arrows* represent the relevant (non-)dominance relations between subpatterns

Consider the following patterns and their parse trees in Fig. 2 (we write ?*x* ≠ ?*y* for ¬(?*x* = ?*y*)):

6 $$\begin{array}{@{}rcl@{}} & ((?x, a, a)~{{\mathsf{OPT}}}~((?x, b, ?y)~{{\mathsf{OPT}}}~(?y, c, ?z)))~{{\mathsf{OPT}}}~(?x, d, ?z), \end{array} $$

7 $$\begin{array}{@{}rcl@{}} & ((?x, a, a)~{{\mathsf{OPT}}}~(?x, d, ?z))~{{\mathsf{OPT}}}~((?x, b, ?y)~{{\mathsf{OPT}}}~(?y, c, ?z)), \end{array} $$

8 $$\begin{array}{@{}rcl@{}} & (((?u, f, ?v)~{{\mathsf{OPT}}}~(?u, g, ?w)) \; {{\mathsf{FILTER}}} \; ?v \neq {?w})~{{\mathsf{OPT}}}~(?u, h, ?s), \end{array} $$

9 $$\begin{array}{@{}rcl@{}} & (?u, h, ?s)~{{\mathsf{OPT}}}~(((?u, f, ?v)~{{\mathsf{OPT}}}~(?u, g, ?w)) \; {{\mathsf{FILTER}}} \; ?v \neq {?w}). \end{array} $$

Pattern (6) is not well-designed because of variable ?*z*, but is weakly well-designed since the occurrence of (?*y*, *c*, ?*z*) dominates (?*x*, *d*, ?*z*). However, the similar pattern (7) is not weakly well-designed because the occurrence of the inner O P T-pattern with the second occurrence of ?*z* does not dominate the first. Pattern (8) is weakly well-designed since the F I L T E R-pattern (which is not dominated by the inner O P T-pattern) is top-level, but pattern (9) is not, because of variable ?*w* in a non-top-level F I L T E R.

### **Proposition 1**

*Checking whether a* U N I O N *-free* *pattern P belongs to the fragment* $\mathcal {P}_{\text {wwd}}$ *can be done in time* *O*(|*P*|^2^)*,* *where* |*P*| *is the length of the string representation of P.*

### *Proof*

**Fig. 3:**
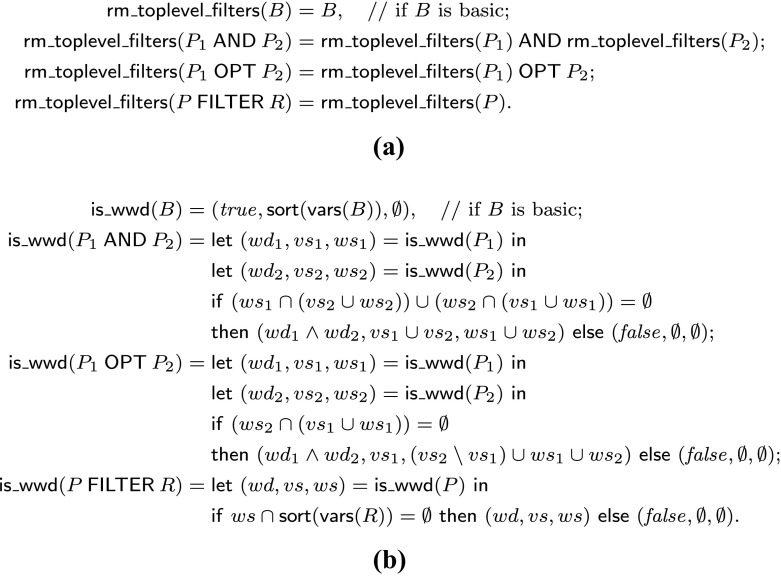
Procedures **a** r m_t o p l e v e l_fi l t e r *s* and **b** *i* *s*_*w* *w* *d*

First note that a U N I O N-free pattern *P* is weakly well-designed if and only if so is the pattern r m_t o p l e v e l_fi l t e r *s*(*P*), which is obtained from *P* by removing all top-level occurrences of filters. The operation r m_t o p l e v e l_fi l t e r *s* can be implemented in linear time by the recursive procedure in Fig. 3a.

Next consider the recursive procedure *i* *s*_*w* *w* *d* in Fig. 3b, where *s* *o* *r* *t*(*S*) denotes a sorted, repetition-free list representation of a set *S*.

Given a U N I O N-free pattern *P* without top-level filters, it is easily seen that *i* *s*_*w* *w* *d*(*P*) returns a tuple of the form (*t* *r* *u* *e*, *v* *s*, *w* *s*) if and only if *P* is weakly well-designed, where *ws* is the sorted list of “unguarded” variables in *P*, that is, variables occurring in the second argument of an O P T-subpattern *P* ^′^ of *P* but not in the first argument of *P* ^′^, and *v* *s* = *s* *o* *r* *t*(*v* *a* *r* *s*(*P*))∖*w* *s*. Procedure *i* *s*_*w* *w* *d* can be implemented in quadratic time since *s* *o* *r* *t* (which may take time *O*(*n* log *n*)) is only applied to atomic subexpressions and set operations on sorted lists take linear time. □

## OPT-FILTER-Normal Form and Constraint Pattern Trees

One of the key properties of wd-patterns is that they can always be converted to a so-called O P T-normal form, in which all A N D- and F I L T E R-subpatterns are O P T-free [33]. Also, F I L T E R-free patterns in O P T-normal form can be naturally represented as trees of a special form [27, 35], which give a good intuition for the evaluation and optimisation of such patterns. In this section, we show that these notions can be generalised to wwd-patterns.

### **Definition 5**

A pattern $P\in \mathcal {P}$ is in O P T- F I L T E R-*normal form* (or O F-*normal form* for short) if it adheres to the grammar

$$\begin{array}{lll} P &::= & F ~\mid~ (P~ {{\mathsf{FILTER}}} ~R) ~\mid~ (P~{{\mathsf{OPT}}}~S),\\ S & ::= & F ~\mid~ (S~{{\mathsf{OPT}}}~S), \\ F & ::= & (B~ {{\mathsf{FILTER}}} ~R), \end{array} $$

where *B* ranges over basic patterns and *R* over filter constraints.

In other words, the parse tree of a pattern in O F-normal form can be stratified as follows:

1. (occurrences of) basic patterns as the bottom layer,
2. a F I L T E R on top of each basic pattern as the middle layer,
3. a combination of O P T and F I L T E R as the top layer;

moreover, each occurrence of a F I L T E R-pattern in the top layer is top-level (according to Definition 3). Note that our normal form is A N D-free: all conjunctions are expressed via basic patterns.

### *Example 2*

None of the four patterns in Example 1 are in O F-normal form. However, the first three of them can be easily normalised by replacing each triple *t* with *t* ^⊤^, where *P* ^⊤^ is an abbreviation of *P* F I L T E R ⊤ for a pattern *P*. Also, compare the pattern

10 $$\begin{array}{@{}rcl@{}} &&(((?x, a, a)^{\top}~{{\mathsf{OPT}}}~(?x, b, ?y)^{\top})~{{\mathsf{OPT}}}~{} ((?x, b, ?z)^{\top}~{{\mathsf{OPT}}}~(?z, c, ?u)^{\top})) \\ && {{\mathsf{FILTER}}}~ ?u \neq {?x},\\ \end{array} $$

which is in O F-normal form, with the very similar pattern

$$\begin{array}{@{}rcl@{}} && ((?x, a, a)^{\top}~{{\mathsf{OPT}}}~(?x, b, ?u)^{\top})~{{\mathsf{OPT}}}\\ &&(((?x, b, ?z)^{\top}~{{\mathsf{OPT}}}~ (?z, c, ?u)^{\top}) \; {{\mathsf{FILTER}}}\: ?u \neq {?z}), \end{array} $$

which is not, because the outer F I L T E R is in the right argument of the outermost O P T.

As shown by Letelier et al. [27], F I L T E R-free patterns in O P T-normal form can be represented by means of so-called pattern trees. We next show that this representation can be naturally extended to patterns in O F-normal form.

### **Definition 6**

Let *P* be a pattern in O F-normal form. The *constraint pattern tree (CPT)* $\mathcal {T}(P)$ of *P* is the directed, ordered, labelled, rooted tree recursively constructed as follows (in this definition we abuse notation and confuse patterns and their occurrences; strictly speaking, we create a fresh sub-tree for each occurrence, so the resulting object is always a tree):

1. if *B* is a basic pattern then $\mathcal {T}(B~ {{\mathsf {FILTER}}}~ R)$ is a single node *v* labelled by the pair (*B*, *R*);
2. if *P* ^′^ is not a basic pattern then $\mathcal {T}(P^{\prime }~ {{\mathsf {FILTER}}} ~R)$ is obtained by adding a *special* node labelled by *R* as the last child of the root of $\mathcal {T}(P^{\prime })$;
3. $\mathcal {T}(P_{1}~{{\mathsf {OPT}}}~P_{2})$ is the tree obtained from $\mathcal {T}(P_{1})$ and $\mathcal {T}(P_{2})$ by adding the root of $\mathcal {T}(P_{2})$ as the last child of the root of $\mathcal {T}(P_{1})$.

**Fig. 4:**
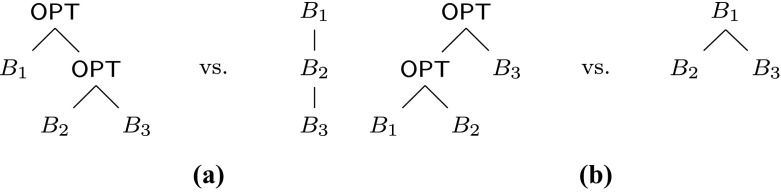
Parse trees vs. constraint pattern trees for patterns **a** *B*_1_ O P T (*B*_2_ O P T *B*_3_) and **b** (*B*_1_ O P T *B*_2_) O P T *B*_3_, with *B*_1_, *B*_2_, and *B*_3_ basic patterns

**Fig. 5:**
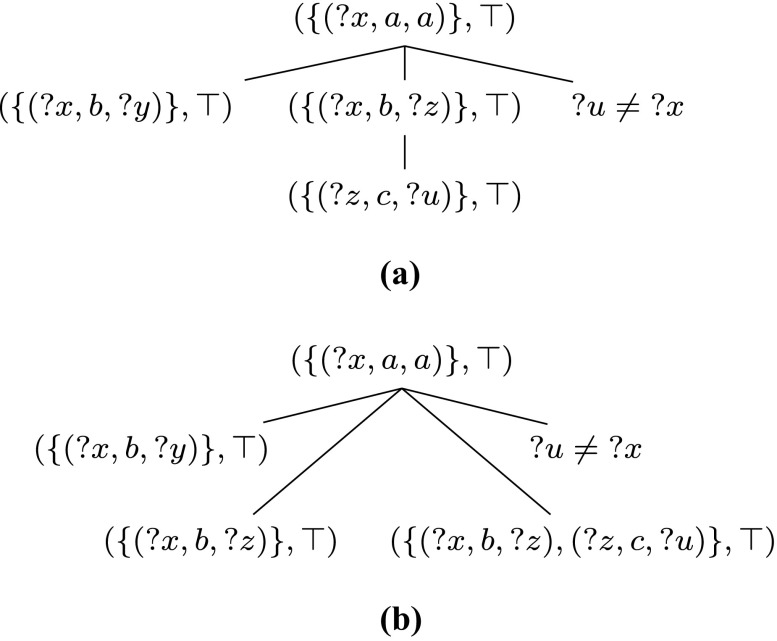
Constraint pattern trees of **a** (((?*x*, *a*, *a*)^⊤^ O P T (?*x*, *b*, ?*y*)^⊤^) O P T ((?*x*, *b*, ?*z*)^⊤^ O P T (?*z*, *c*, ?*u*)^⊤^)) F I L T E R ?*u* ≠ ?*x* (i.e., pattern (10)) and **b** equivalent pattern in “flat” form (13)

By definition, there is a one-to-one correspondence between patterns in O F-normal form and CPTs. Hence, such trees can be seen as a convenient representation of patterns in O F-normal form. Unlike parse trees, which represent the syntactic shape of patterns, CPTs show the semantic structure of O P T and F I L T E R nesting. Figure 4 shows how O P T nestings of types (Opt-R) and (Opt-L) are represented in both formats. Note that CPTs treat different F I L T E R-subpatterns differently: if the filter is over a basic pattern, the constraint of the F I L T E R is paired with this pattern; however, if the filter is over an O P T-subpattern, then the constraint is represented by a separate special node. Moreover, since in the second case the F I L T E R-pattern must be top-level, special nodes can only occur in CPTs as children of the root. For instance, the CPT of the example pattern (10) is given in Fig. 5a.

### **Proposition 2**

*Let P be a pattern in* O F *-normal* *form. Then every special node in* $\mathcal {T}(P)$ *is a child of the root.*

### *Proof*

Let *v* be a special node in $\mathcal {T}(P)$. Then *v* is obtained from a subpattern *P* ^′^ F I L T E R *R* where *P* ^′^ is not basic. Hence, by definition of the O F-normal form, *P* must have the form

$$(\dots((P^{\prime}~{{\mathsf{FILTER}}}~ R)~{{\mathsf{OPT}}}~ S_{1})\dots)~{{\mathsf{OPT}}}~ S_{n} $$

(for some *n* ≥ 0) where *S*_1_, …, *S*_*n*_ contain only F I L T E R-subpatterns over basic patterns. Thus, the root of $\mathcal {T}(P^{\prime })$ is also the root of $\mathcal {T}(P)$, and the claim follows. □

Next we show that each wwd-pattern can be converted to O F-normal form and hence can be represented by a CPT. To prove this statement we make use of a number of equivalences. Formally, a pattern *P*_1_ is *equivalent* to a pattern *P*_2_ (written *P*_1_ ≡ *P*_2_) if [[*P*_1_]]_*G*_ = [[*P*_2_]]_*G*_ holds for any graph *G*. There are several equivalences, such as associativity and commutativity of A N D, as well as filter decompositions, such as *P* F I L T E R (*R*_1_ ∧ *R*_2_) ≡ (*P* F I L T E R *R*_1_) F I L T E R *R*_2_, which hold for all patterns (see [38] for an extensive list). Moreover, the key equivalences used in [33] for normalising wd-patterns can easily be adapted to serve our needs.

### **Proposition 3**

*Let* *P*_1_, *P*_2_, *P*_3_ *be patterns and R a filter constraint such that* *v* *a* *r* *s*(*P*_2_) ∩ *v* *a* *r* *s*(*P*_3_) ⊆ *v* *a* *r* *s*(*P*_1_) *and* *v* *a* *r* *s*(*P*_2_) ∩ *v* *a* *r* *s*(*R*) ⊆ *v* *a* *r* *s*(*P*_1_)*.* *Then the following equivalences hold:*

$$\begin{array}{c} (P_{1}~{{\mathsf{OPT}}} ~P_{2})~{{\mathsf{AND}}}~ P_{3} \equiv (P_{1}~{{\mathsf{AND}}} ~P_{3})~{{\mathsf{OPT}}}~ P_{2}, \\ (P_{1}~{{\mathsf{OPT}}}~ P_{2})~{{\mathsf{FILTER}}}~ R \equiv (P_{1}~{{\mathsf{FILTER}}}~ R)~{{\mathsf{OPT}}}~ P_{2}. \end{array} $$

### *Proof*

Both equivalences are essentially shown in [33]. While stated for well-designed patterns, the proof only exploits the properties *v* *a* *r* *s*(*P*_2_) ∩ *v* *a* *r* *s*(*P*_3_) ⊆ *v* *a* *r* *s*(*P*_1_) and *v* *a* *r* *s*(*P*_2_) ∩ *v* *a* *r* *s*(*R*) ⊆ *v* *a* *r* *s*(*P*_1_), which are satisfied not only by well-designed patterns, but also by weakly well-designed patterns □

Since all the equivalences preserve weak well-designedness, we obtain the desired result.

### **Proposition 4**

*Each wwd-pattern P is equivalent to a wwd-pattern in* O F *-normal* *form of size* *O*(|*P*|)*.*

### *Proof*

We call a pattern *P pre-normal* if it adheres to the grammar that is the same as the one in Definition 5 except that the category *F* is extended as follows:

$$F::= B ~\mid~ (F\,{{\mathsf{FILTER}}}\,R) ~\mid~ (F\,{{\mathsf{AND}}}\,F). $$

Given a pattern *P*, let ||*P*|| be the sum of the sizes of all A N D-subpatterns and all F I L T E R-subpatterns of *P* (where different occurrences of each pattern are counted separately). Consider a wwd-pattern *P* that is not pre-normal. Then *P* contains a subpattern *P* ^′^ of one of the following two forms (modulo commutativity of A N D): (*P*_1_ O P T *P*_2_) A N D *P*_3_ and (*P*_1_ O P T *P*_2_) F I L T E R *R* with *P* ^′^ not top-level. In both cases we can rewrite *P* to a pattern *S* not increasing |⋅| and strictly decreasing ||⋅|| as follows.

- Let *P* ^′^ = (*P*_1_ O P T *P*_2_) A N D *P*_3_. Since *P* is weakly well-designed and the occurrence of *P*_3_ is not dominated by the occurrence of *P*_1_ O P T *P*_2_, we have *v* *a* *r* *s*(*P*_3_) ∩ *v* *a* *r* *s*(*P*_2_) ⊆ *v* *a* *r* *s*(*P*_1_). Therefore, using the first equivalence in Proposition 3, we can rewrite *P* to a pattern *S* by replacing *P* ^′^ with (*P*_1_ A N D *P*_3_) O P T *P*_2_. Moreover, we have |*P*| = |*S*| and ||*P*|| > ||*S*||.
- Let *P* ^′^ = (*P*_1_ O P T *P*_2_) F I L T E R *R* where the occurrence of *P* ^′^ is not top-level. Since *P* is weakly well-designed, we then have *v* *a* *r* *s*(*R*) ∩ *v* *a* *r* *s*(*P*_2_) ⊆ *v* *a* *r* *s*(*P*_1_), and thus, with the second equivalence in Proposition 3, we can rewrite *P* to a pattern *S* by replacing *P* ^′^ with (*P*_1_ F I L T E R *R*) O P T *P*_2_. Moreover, we have |*P*| = |*S*| and ||*P*|| > ||*S*||.

Since this rewriting strictly decreases ||⋅||, its repeated application to *P* terminates and yields a pre-normal pattern *S* equivalent to *P* with |*S*| = |*P*|.

Finally, *S* can be transformed to O F-normal form by replacing every occurrence of an A N D- F I L T E R combination of basic patterns by *B* F I L T E R *R* where *B* consists of all triples in the basic patterns and *R* is a conjunction of all the filter conditions (if there are no filters in the combination, then *R* is ⊤). Clearly, this transformation is equivalence-preserving and linear in |*S*|. □

Relying on this proposition, in the rest of the paper we silently assume that all wwd-patterns are in O F-normal form and hence can be represented by CPTs.

We next transfer the notion of weak well-designedness to CPTs. Given a pattern *P* in O F-normal form, let ≺ be the strict topological sorting of the nodes in $\mathcal {T}(P)$ computed by a depth first search traversal visiting the children of a node according to their ordering (i.e., *v* ≺ *u* holds if *v* is visited before *u*).

### **Lemma 1**

*Let P be a pattern in* O F *-normal* *form and* *P* ^′^ = *P*_1_ O P T *P*_2_ *be a subpattern of P. Then* *v* ≺ *w* *for every two nodes v, w in* $\mathcal {T}(P)$ *such that v is in the subtree of* $\mathcal {T}(P)$ *corresponding to* *P*_1_ *and w is in the subtree corresponding to* *P*_2_ *.*

### *Proof*

The claim follows since $\mathcal {T}(P^{\prime })$ is constructed by attaching $\mathcal {T}(P_{2})$ as the last child to the root of $\mathcal {T}(P_{1})$. □

In the following proposition, *v* *a* *r* *s*(*u*) for a node *u* of a CPT stands for the set of all variables in the label of *u*.

### **Proposition 5**

*A pattern P in* O F *-normal* *form is weakly well-designed if and only if, for each edge* (*v*, *u*) *with non-special u in the CPT* $\mathcal {T}(P)$ *,* *every variable* ?*x* ∈ *v* *a* *r* *s*(*u*) ∖ *v* *a* *r* *s*(*v*) *occurs only in nodes w such that* *v* ≺ *w* *.* *The pattern is well-designed if and only if for every variable* ?*x* *in P the set of all nodes v in* $\mathcal {T}(P)$ *with* ?*x* ∈ *v* *a* *r* *s*(*v*) *is connected.*

### *Proof*

For the forward direction of the first statement, suppose *P* is weakly well-designed. We proceed by induction on the structure of *P* and consider the following cases.

- Let *P* = *B* F I L T E R *R* where *B* is basic. Then the claim is vacuous.
- Let *P* = *P*_1_ F I L T E R *R* where *P*_1_ is not basic. By the inductive hypothesis, the claim holds for $\mathcal {T}(P_{1})$. Moreover, $\mathcal {T}(P)$ differs from $\mathcal {T}(P_{1})$ only in the special node labelled with *R*, and the claim follows by Proposition 2.
- Let *P* = *P*_1_ O P T *P*_2_. By the inductive hypothesis, the claim holds for $\mathcal {T}(P_{1})$ and $\mathcal {T}(P_{2})$. Thus, by Lemma 1, it suffices to show that for every edge (*v*, *u*) in $\mathcal {T}(P_{2})$ (with non-special *u* by definition), no variable ?*x* ∈ *v* *a* *r* *s*(*u*) ∖ *v* *a* *r* *s*(*v*) occurs in $\mathcal {T}(P_{1})$. Suppose for contradiction that this property is violated for some (*v*, *u*) and ?*x*. Then *P*_2_ has a subpattern $P^{\prime }=P^{\prime }_{1}~{{\mathsf {OPT}}}~P^{\prime }_{2}$ such that $\mathcal {T}(P^{\prime }_{1})$ is a subtree of $\mathcal {T}(P)$ rooted at *v* and $\mathcal {T}(P^{\prime }_{2})$ is the complete subtree of $\mathcal {T}(P)$ rooted at *u*. Moreover, ?*x* occurs in *P*_1_, and thus outside *P* ^′^. Since all F I L T E R-subpatterns in *P* are safe, we can assume without loss of generality that the occurrence of ?*x* in *P*_1_ is not in a filter constraint. However, this contradicts the assumption that *P* is weakly well-designed since the occurrence of ?*x* in *P*_1_ is not dominated by the occurrence of *P* ^′^.

For the backward direction of the first claim, suppose *P* is not a wwd-pattern. Then *P* has a subpattern $P^{\prime }=P^{\prime }_{1}~{{\mathsf {OPT}}}~ P^{\prime }_{2}$, with *v* the root of $\mathcal {T}(P^{\prime })$ in $\mathcal {T}(P)$ and *u* the child of *v* corresponding to $\mathcal {T}(P^{\prime }_{2})$, and a variable $?x\in {\mathsf {vars}(P^{\prime }_{2})}\setminus {\mathsf {vars}(P^{\prime }_{1})}$ such that ?*x* ∈ *v* *a* *r* *s*(*u*) and, for some subpattern *P*_1_ O P T *P*_2_ of *P*, ?*x* occurs in *P*_1_ and *P* ^′^ occurs in *P*_2_. Since $?x\in {\mathsf {vars}(P^{\prime }_{2})}\setminus {\mathsf {vars}(P^{\prime }_{1})}$ and ?*x* ∈ *v* *a* *r* *s*(*u*), we have ?*x* ∈ *v* *a* *r* *s*(*u*) ∖*v* *a* *r* *s*(*v*). Thus, by Lemma 1, we have *v* ⊀ *w*, where *w* is a node in $\mathcal {T}(P_{1})$ with an occurrence of ?*x*.

The second claim can be proved analogously. □

Note that if a pattern is F I L T E R-free, its O F-normal form coincides with the O P T-normal form in [33] (modulo tautological filters), and its CPT is the pattern tree from [27, 35]. In fact, the second part of Proposition 5 generalises an observation from [27] to the case with filters. An important difference to pattern trees is that in our case the order of children of a node is semantically relevant since wwd-patterns do not satisfy the equivalence

11 $$ (P_{1}~{{\mathsf{OPT}}}~ P_{2})~{{\mathsf{OPT}}}~P_{3} ~\equiv~ (P_{1}~{{\mathsf{OPT}}}~ P_{3})~{{\mathsf{OPT}}}~ P_{2} . $$

This equivalence, established in [32], holds whenever (*v* *a* *r* *s*(*P*_2_) ∩ *v* *a* *r* *s*(*P*_3_)) ⊆ *v* *a* *r* *s*(*P*_1_), which is always the case for wd-patterns but not for wwd-patterns, as can be seen on query (2).

We conclude this section with a property that is unique to wwd-patterns: each wwd-pattern is equivalent to a pattern whose corresponding CPT has depth one.

### **Definition 7**

A pattern in $\mathcal {P}$ is in *depth-one normal form* if it has the structure

12 $$ ({\cdots} ((B ~{\mathsf{op}}_{1}~ S_{1})~ {\mathsf{op}}_{2}~ S_{2}) {\cdots} )~ {\mathsf{op}}_{n}~ S_{n}, $$

where *B* is a basic pattern and each *o* *p*_*i*_ *S*_*i*_, 1 ≤ *i* ≤ *n*, is either O P T (*B*_*i*_ F I L T E R *R*_*i*_) with *B*_*i*_ a basic pattern and *R*_*i*_ a filter constraint, or just F I L T E R *R*_*i*_.

To show that each wwd-pattern can be brought to this form, we exploit the following observation in [33].

### **Lemma 2** (Pérez et al. [33])

*Let P be a pattern in* $\mathcal {P}$ *, G a graph, and* *μ*_1_ *,* *μ*_2_ *two mappings in* [[*P*]]_*G*_ *.* *Then* *μ*_1_ ∼ *μ*_2_ *if and only if* *μ*_1_ = *μ*_2_ *.*

This lemma allows us to prove the following crucial equivalence.

### **Proposition 6**

*For patterns* *P*_1_ *,* *P*_2_ *and* *P*_3_ *in* $\mathcal {P}$ *with* *v* *a* *r* *s*(*P*_1_) ∩ *v* *a* *r* *s*(*P*_3_) ⊆ *v* *a* *r* *s*(*P*_2_)*it holds that*

13 $$ P_{1}~{{\mathsf{OPT}}}~(P_{2}~{{\mathsf{OPT}}}~ P_{3}) ~\equiv~ (P_{1}~{{\mathsf{OPT}}}~ P_{2})~{{\mathsf{OPT}}}~(P_{2}~{{\mathsf{AND}}}~P_{3}). $$

### *Proof*

We first show that any solution to the left hand side is also a solution to the right hand side. Let *G* be a graph and let *μ* ∈ [[*P*_1_ O P T (*P*_2_ O P T *P*_3_)]]_*G*_. We distinguish three cases.

- Let *μ* ∈ [[*P*_1_]]_*G*_ ⋈ ([[*P*_2_]]_*G*_ ⋈ [[*P*_3_]]_*G*_). Then, by Lemma 2, we have [[*P*_2_]]_*G*_ = [[*P*_2_]]_*G*_ ⋈ [[*P*_2_]]_*G*_. Consequently, *μ* ∈ ([[*P*_1_]]_*G*_ ⋈ [[*P*_2_]]_*G*_) ⋈ ([[*P*_2_]]_*G*_ ⋈ [[*P*_3_]]_*G*_), and the claim follows.
- Let *μ* ∈ [[*P*_1_]]_*G*_ ⋈ ([[*P*_2_]]_*G*_ ∖ [[*P*_3_]]_*G*_). Then *μ* = *μ*_1_ ∪ *μ*_2_ such that *μ*_1_ ∈ [[*P*_1_]]_*G*_, *μ*_2_ ∈ [[*P*_2_]]_*G*_, and for every *μ*_3_ ∈ [[*P*_3_]]_*G*_, *μ*_2_ ≁ *μ*_3_. Since every mapping in [[*P*_2_ A N D *P*_3_]]_*G*_ is an extension of some mapping in [[*P*_3_]]_*G*_, no mapping in [[*P*_2_ A N D *P*_3_]]_*G*_ is compatible with *μ*_2_, and hence with *μ*. Therefore, *μ* ∈ ([[*P*_1_]]_*G*_ ⋈ [[*P*_2_]]_*G*_) ∖ [[*P*_2_ A N D *P*_3_]]_*G*_, and the claim follows.
- Let *μ* ∈ [[*P*_1_]]_*G*_ ∖ [[*P*_2_ O P T *P*_3_]]_*G*_. Then *μ* ∈ [[*P*_1_]]_*G*_ and is incompatible with any mapping in [[*P*_2_ O P T *P*_3_]]_*G*_. Moreover, since *v* *a* *r* *s*(*P*_1_) ∩ *v* *a* *r* *s*(*P*_3_) ⊆ *v* *a* *r* *s*(*P*_2_), *μ* is incompatible with any mapping in [[*P*_2_]]_*G*_, and consequently also with any mapping in [[*P*_2_ A N D *P*_3_]]_*G*_. Therefore, *μ* ∈ ([[*P*_1_]]_*G*_ ∖ [[*P*_2_]]_*G*_) ∖ [[*P*_2_ A N D *P*_3_]]_*G*_, and the claim follows.

For the other direction, suppose *μ* ∈ [[(*P*_1_ O P T *P*_2_) O P T (*P*_2_ A N D *P*_3_)]]_*G*_. We distinguish three cases.

- Let *μ* ∈ ([[*P*_1_]]_*G*_ ⋈ [[*P*_2_]]_*G*_) ⋈ ([[*P*_2_]]_*G*_ ⋈ [[*P*_3_]]_*G*_). Then, by Lemma 2, we have *μ* ∈ [[*P*_1_]]_*G*_ ⋈ ([[*P*_2_]]_*G*_ ⋈ [[*P*_3_]]_*G*_), and the claim follows.
- Let *μ* ∈ ([[*P*_1_]]_*G*_ ⋈ [[*P*_2_]]_*G*_) ∖ ([[*P*_2_]]_*G*_ ⋈ [[*P*_3_]]_*G*_). Then *μ* = *μ*_1_ ∪ *μ*_2_ such that *μ*_1_ ∈ [[*P*_1_]]_*G*_, *μ*_2_ ∈ [[*P*_2_]]_*G*_, and *μ* is incompatible with every mapping in [[*P*_2_]]_*G*_ ⋈ [[*P*_3_]]_*G*_. Since *v* *a* *r* *s*(*P*_1_) ∩ *v* *a* *r* *s*(*P*_3_) ⊆ *v* *a* *r* *s*(*P*_2_), this implies that [[*P*_2_]]_*G*_ ⋈ [[*P*_3_]]_*G*_ is empty, that is, *μ*_2_ is incompatible with every mapping in [[*P*_3_]]_*G*_. Therefore, *μ*_2_ ∈ [[*P*_2_]]_*G*_ ∖ [[*P*_3_]]_*G*_, and thus *μ* ∈ [[*P*_1_]]_*G*_ ⋈ ([[*P*_2_]]_*G*_ ∖ [[*P*_3_]]_*G*_). The claim follows.
- Let *μ* ∈ [[*P*_1_]]_*G*_ ∖ [[*P*_2_]]_*G*_. Since every mapping in [[*P*_2_ O P T *P*_3_]]_*G*_ extends a mapping in [[*P*_2_]]_*G*_, we have that *μ* ∈ [[*P*_1_]]_*G*_ ∖ [[*P*_2_ O P T *P*_3_]]_*G*_, and the claim follows.

□

Applied from left to right, equivalence (13) preserves weak well-designedness (but not well-designedness). Each such application transforms a weakly well-designed O P T nesting of type (Opt-R) to a nesting of type (Opt-L), decreasing the depth of the CPT.

### **Corollary 1**

*Every wwd-pattern is equivalent to a wwd-pattern in depth-one normal form.*

For instance, pattern (10) is equivalent to the pattern

$$\begin{array}{@{}rcl@{}} &&((((?x, a, a)^{\top}~{{\mathsf{OPT}}}~(?x, b, ?y)^{\top})~{{\mathsf{OPT}}}~(?x, b, ?z)^{\top})~{{\mathsf{OPT}}} {} \\ && \{(?x, b, ?z),(?z, c, ?u)\}^{\top})~ {{\mathsf{FILTER}}}~ {} ?u \neq {?x}, \end{array} $$

represented by the CPT in Fig. 5b. Such “flat” patterns are attractive in practice because of their regular structure. However, “flattening” a pattern can incur an exponential blow-up in size. Hence, in the rest of the paper we consider arbitrary wwd-patterns in O F-normal form rather than restricting our attention to depth-one-normal patterns.

## Evaluation of wwd-Patterns

In this section, we look at the query answering problem for wwd-patterns and their extensions with union and projection. We show that in all three cases, complexity remains the same as for wd-patterns. To obtain these results, we develop several new techniques.

Formally, we look at the following decision problem for a given SPARQL fragment $\mathcal {L}$. 
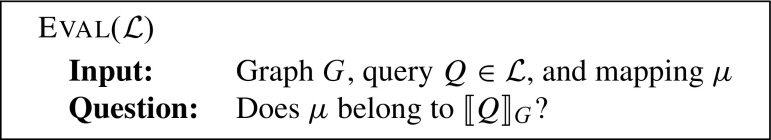


It is known that $\textsc {Eval}({\mathcal {U}})$ for general patterns $\mathcal {U}$ is PSpace-complete [33], and the result easily propagates to queries with projection (i.e., $\mathcal {S}$) [27]. For wd-patterns, the evaluation problem is coNP-complete, and can be solved by exploiting the following idea of [27].

Suppose we are given a wd-pattern *P* in O P T-normal form (for simplicity, assume that *P* is F I L T E R-free), a graph *G*, and a mapping *μ*. First, we look for a subtree of $\mathcal {T}(P)$ that includes the root of $\mathcal {T}(P)$, contains precisely the variables in *d* *o* *m* *μ*, and “matches” *G* under *μ* (i.e., images of all its triples under *μ* are contained in *G*). This is doable in polynomial time. If such a subtree does not exist, then *μ* cannot be a solution. Otherwise, the subtree witnesses that *μ* is a part of a solution to *P*. Finally, to verify that *μ* is a complete solution, we need to check that the subtree is maximal, that is, cannot be extended to any more nodes in $\mathcal {T}(P)$ with a match in *G*. There are linearly many such nodes to check, and each check can be performed in coNP. So, the overall algorithm runs in coNP.

Inspired by this idea, we next show that the low evaluation complexity of wd-patterns transfers to wwd-patterns by developing a coNP algorithm for Eval(${\mathcal {P}_{\text {wwd}}}$).

Let *P* be a wwd-pattern in O F-normal form. An *r-subtree* of $\mathcal {T}(P)$ is a subtree containing the root of $\mathcal {T}(P)$ and all its special children. Every r-subtree $\mathcal {T}(P^{\prime })$ of $\mathcal {T}(P)$ is also a CPT representing a wwd-pattern *P* ^′^ that can be obtained from *P* by dropping the right arguments of some O P T-subpatterns (a transformation known from [33]). A *child* of an r-subtree $\mathcal {T}(P^{\prime })$ of $\mathcal {T}(P)$ is a node in $\mathcal {T}(P)$ that is not contained in $\mathcal {T}(P^{\prime })$ but whose parent is.

### **Definition 8**

A mapping *μ* is a *potential partial solution* (or *pp-solution* for short) to a wwd-pattern *P* over a graph *G* if there is an r-subtree $\mathcal {T}(P^{\prime })$ of $\mathcal {T}(P)$ such that *d* *o* *m*(*μ*) = *v* *a* *r* *s*(*P* ^′^), *μ*(t r i p l e s(*P* ^′^)) ⊆ *G*, and *μ* ⊧ *R* for the constraint *R* of each ordinary node in $\mathcal {T}(P^{\prime })$.

A pp-solution *μ* to *P* over *G* can be witnessed by several r-subtrees. However, the union of such r-subtrees is also a witness. Hence, there exists a unique maximal witnessing r-subtree, denoted $\mathcal {T}(P_{\mu })$, with *P*_*μ*_ being the corresponding wwd-pattern.

Potential partial solutions generalise “partial solutions” as defined in [33] for wd-patterns. There, every “partial solution” is either a solution or can be extended to one. This is not the case for wwd-patterns. While every solution is clearly a pp-solution, not every pp-solution can be extended to a real one. Real solutions may not just extend pp-solutions by assigning previously undefined variables but can also override variable bindings established in some node *v* of $\mathcal {T}(P_{\mu })$ by extending to a child of $\mathcal {T}(P_{\mu })$ that precedes *v* according to the order ≺.

An additional complication is the presence of non-well-designed top-level filters. Note that pp-solutions are only required to satisfy the constraints of ordinary nodes in the corresponding CPT, thus ignoring top-level filters. Indeed, requiring pp-solutions to satisfy constraints of top-level filters would be too strong since real solutions do not generally satisfy this property, as demonstrated by the following example.

### *Example 3*

Consider the graph *G* = {(1, *a*, 1),(3, *a*, 3)} and wwd-pattern

$$P = (({(?x, a, 1)}~{{\mathsf{OPT}}}~{(?y, a, 2)})~{{\mathsf{FILTER}}}\,\neg bound(?y))~{{\mathsf{OPT}}}~{(?y, a, 3)}. $$

The mapping *μ* = {?*x* ↦ 1, ?*y* ↦ 3} is a solution to *P* over *G*, but *μ* ⊭ ¬*b* *o* *u* *n* *d*(?*y*).

We now present a characterisation of solutions for wwd-patterns in terms of pp-solutions that (a) takes into account that not every pp-solution can be extended to a real solution and (b) ensures correct treatment of non-well-designed top-level filters. For this we need some more notation. Given a wwd-pattern *P*, a node *v* in $\mathcal {T}(P)$, a graph *G*, and a pp-solution *μ* to *P* over *G*, let *μ*|_*v*_ be the projection *μ*|_*X*_ of *μ* to the set *X* of all variables appearing in nodes *u* of $\mathcal {T}(P_{\mu })$ such that *u* ≺ *v*. A mapping *μ*_1_ is *subsumed* by a mapping *μ*_2_ (written $\mu _{1} \sqsubseteq \mu _{2}$) if *μ*_1_ ∼ *μ*_2_ and *d* *o* *m*(*μ*_1_) ⊆ *d* *o* *m*(*μ*_2_) (this notion is from [5, 33]).

Intuitively, a pp-solution *μ* needs to satisfy two conditions to be a real solution to a wwd-pattern *P*. First, *μ*|_*v*_ (as opposed to *μ* for wd-patterns) must be non-extendable to *v* for any child *v* of $\mathcal {T}(P_{\mu })$. Indeed, if such an extension exists, then it is either possible to provide bindings for some variables that are undefined in *μ*, or some variables from *d* *o* *m*(*μ*) can be assigned different values of higher “priority” than the corresponding values in *μ*. Second, every top-level filter *R* labelling a node *s* needs to be satisfied by *μ*|_*s*_, which is precisely the part of *μ* bound by the subpattern of *P* that is paired with *R* in the F I L T E R-pattern. The following lemma formalises this intuition.

### **Lemma 3**

*A mapping* *μ* *is a solution to a wwd-pattern P over a graph G if and only if*

1. *μ* *is a pp-solution to P over G;*
2. *for every child v of* $\mathcal {T}(P_{\mu })$ *labelled with* (*B*, *R*) *there is no* *μ* ^′^ *such that* $\mu |_{v} \sqsubseteq \mu ^{\prime }$ *,* *μ* ^′^ ⊧ *R* *,* *and* *μ* ^′^(*B*) ⊆ *G* *;*
3. *μ*|_*s*_ ⊧ *R* *for every special node s in* $\mathcal {T}(P)$ *labelled with R.*

### *Proof*

In this proof we write $\mathcal {T}_{v}$ for the complete subtree of a CPT $\mathcal {T}$ rooted at a node *v* (i.e., the subtree over all the descendants of *v* including *v* itself) and $\mathcal {T}_{\prec v}$ for the subtree of $\mathcal {T}$ consisting of all nodes u such that *u* ≺ *v*.

For the forward direction, suppose *μ* is a solution to *P* over *G*. Clearly, *μ* is a pp-solution to *P* over *G*, so it suffices to show that conditions 2 and 3 hold.

For condition 2, assume for contradiction that *v* is a child of $\mathcal {T}(P_{\mu })$ labelled with (*B*, *R*) and *μ* ^′^ a mapping such that $\mu |_{v}\sqsubseteq \mu ^{\prime }$, *μ* ^′^ ⊧ *R*, and *μ* ^′^(*B*) ⊆ *G*. Moreover, without loss of generality, let *d* *o* *m*(*μ* ^′^) = *d* *o* *m*(*μ*) ∪ *v* *a* *r* *s*(*B*). Let *u* be the parent of *v* in $\mathcal {T}(P)$, and let $\mathcal {T}$ be the largest subtree of $\mathcal {T}(P)$ that is rooted at *u* and has *v* as the last child of *u*. Then . Moreover, since *u* is contained in $\mathcal {T}(P_{\mu })$, there is a mapping $\mu _{1}\sqsubseteq \mu $ such that $\mu _{1}\in [{\kern -2.3pt}[ \mathcal {T} ]{\kern -2.3pt}]_{G}$. Since *v* is not contained in $\mathcal {T}(P_{\mu })$, we have $\mu _{1}\sqsubseteq \mu |_{v}$ and, since $\mathcal {T}(P_{\mu })$ is the largest r-subtree witnessing *μ*, *μ*_1_ is not compatible with any mapping in $[{\kern -2.3pt}[ \mathcal {T}_{v} ]{\kern -2.3pt}]_{G}$. On the other hand, *μ* ^′^ satisfies the label of *v*, and thus, since $\mathcal {T}_{v}$ contains no top-level filters, *μ* ^′^|_*vars*(*v*)_ can be extended to a mapping of $\mu ^{\prime \prime }\in [{\kern -2.3pt}[ \mathcal {T}_{v} ]{\kern -2.3pt}]_{G}$. Moreover, since *P* is weakly well-designed, ${\mathsf {vars}(\mathcal {T}_{v})}\cap {\mathsf {dom}(\mu |_{v})}\subseteq {\mathsf {vars}(v)}$, and hence *d* *o* *m*(*μ* ^″^) ∩ *d* *o* *m*(*μ*_1_) ⊆ *d* *o* *m*(*μ* ^′^). Thus, since *μ*|_*v*_ is compatible with *μ* ^′^, *μ*_1_ is compatible with *μ* ^″^, in contradiction to the above observation that *μ*_1_ is not compatible with any mapping in $[{\kern -2.3pt}[ \mathcal {T}_{v} ]{\kern -2.3pt}]_{G}$.

For condition 3, let *s* be a special node in $\mathcal {T}(P)$ labelled with *R*. Since *μ* is a solution to *P*, there is some *μ*_1_ ⊆ *μ* such that $\mu _{1}\in [{\kern -2.3pt}[ \mathcal {T}(P)_{\prec s} ]{\kern -2.3pt}]_{G}$ and *μ*_1_ ⊧ *R*. Hence, it suffices to show that *μ*_1_ = *μ*|_*s*_. Clearly, *μ*_1_ ⊆ *μ*|_*s*_ (as *μ*|_*s*_ is the largest mapping compatible with *μ* that can occur in $[{\kern -2.3pt}[ \mathcal {T}(P)_{\prec s} ]{\kern -2.3pt}]_{G}$), so assume for contradiction that there is a variable ?*x* ∈ *d* *o* *m*(*μ*|_*s*_) ∖ *d* *o* *m*(*μ*_1_). Then there is a node in $\mathcal {T}(P_{\mu |_{s}})\cap \mathcal {T}(P)_{\prec s}$ that does not occur in $\mathcal {T}(P_{\mu _{1}})\cap \mathcal {T}(P)_{\prec s}$. This yields a contradiction with $\mu _{1}\in [{\kern -2.3pt}[ \mathcal {T}(P)_{\prec s} ]{\kern -2.3pt}]_{G}$ analogously to the case of condition 2.

For the backward direction, suppose that *μ* satisfies conditions 1–3. We show that *μ* ∈ [[*P*]]_*G*_ by induction on the depth of $\mathcal {T}(P_{\mu })$, that is, the maximal number of edges between the root and a leaf.

For the basis of the induction, let the depth of $\mathcal {T}(P_{\mu })$ be 0, that is, the root *v* of $\mathcal {T}(P)$ be the only node of $\mathcal {T}(P_{\mu })$. We prove the claim by induction on the number *n* of children of *v* in $\mathcal {T}(P)$. If *n* = 0, then *P* = *B* F I L T E R *R* for some basic pattern *B* and filter constraint *R*, and the claim follows since *μ* is a pp-solution to *P* over *G*. For the inductive step, suppose the claim holds for all wwd-patterns *P* ^′^ and mappings *μ* ^′^ satisfying 1–3 provided $\mathcal {T}(P^{\prime }_{\mu ^{\prime }})$ has depth 0 and *n* − 1 children in $\mathcal {T}(P^{\prime })$. Let *P* and *μ* be such that $\mathcal {T}(P_{\mu })$ has depth 0 and n children in $\mathcal {T}(P)$. Let *u* be the last child of (the root *v* of) $\mathcal {T}(P_{\mu })$. Then *μ*|_*u*_ is a pp-solution to $\mathcal {T}(P)_{\prec u}$ that satisfies conditions 2 and 3 since (*μ*|_*u*_)|_*w*_ = *μ*|_*w*_ for every *w* ≺ *u*. Hence, by the inductive hypothesis for the pattern corresponding to $\mathcal {T}(P)_{\prec u}$ and the mapping *μ*|_*u*_, we have $\mu |_{u}\in [{\kern -2.3pt}[ \mathcal {T}(P)_{\prec u} ]{\kern -2.3pt}]_{G}$. We distinguish two cases.

- Let *u* be a special node labelled with *R*. Then it suffices to show that *μ*|_*u*_ ⊧ *R*, which is immediate since *μ*|_*u*_ satisfies condition 3.
- Let *u* be an ordinary node labelled with (*B*, *R*). We know that *u* is not in $\mathcal {T}(P_{\mu })$. Since *v* is in $\mathcal {T}(P_{\mu })$, by condition 2 there is no mapping *μ* ^′^ such that (a) $\mu |_{u}\sqsubseteq \mu ^{\prime }$, (b) *μ* ^′^ ⊧ *R*, and (c) *μ* ^′^(*B*) ⊆ *G*. Since R is safe, it follows that every mapping satisfying (b) and (c) is incompatible with *μ*|_*u*_. Consequently, every mapping in $[{\kern -2.3pt}[ \mathcal {T}(P)_{u} ]{\kern -2.3pt}]_{G}$ is incompatible with *μ*|_*u*_, and hence $\mu =\mu |_{u}\in [{\kern -2.3pt}[ \mathcal {T}(P)_{\prec u} ]{\kern -2.3pt}]_{G}\setminus [{\kern -2.3pt}[ \mathcal {T}(P)_{u} ]{\kern -2.3pt}]_{G}$, as required.

For the outer inductive step, let the claim hold for all *P* ^′^ and *μ* ^′^ with $\mathcal {T}(P^{\prime }_{\mu ^{\prime }})$ of depth *d* − 1, for some *d* > 0. Once again, we show the claim for *P* and *μ* with $\mathcal {T}(P_{\mu })$ of depth *d* by induction on the number *n* of children of the root *v* of $\mathcal {T}(P)$. The basis is vacuous as *v* cannot have 0 children while $\mathcal {T}(P_{\mu })$ has positive depth. The inductive step is the same as for depth 0, except that we have an additional case for the last child *u* of the root *v*.

- Let *u* be an ordinary node labelled with (*B* ^′^, *R* ^′^) that is contained in $\mathcal {T}(P_{\mu })$. Then *μ* = *μ*|_*u*_ ∪ *μ*_2_ where *μ*_2_ is the projection of *μ* to the set of variables occurring in the subtree $\mathcal {T}$ of $\mathcal {T}(P_{\mu })$ rooted at *u* (i.e., $\mathcal {T}=\mathcal {T}(P_{\mu })_{u}$). Since *u* is contained in $\mathcal {T}(P_{\mu })$ and contains no special children, *μ*_2_ is a pp-solution to (the subpattern represented by) $\mathcal {T}(P)_{u}$. Moreover, *μ*_2_ satisfies condition 3 with respect to $\mathcal {T}(P)_{u}$ since $\mathcal {T}(P)_{u}$ contains no special nodes. We next show that *μ*_2_ satisfies condition 2 with respect to $\mathcal {T}(P)_{u}$. Let *w* be a child of $\mathcal {T}$ (in $\mathcal {T}(P)_{u}$) labelled with (*B*, *R*), and assume for contradiction that there is some *μ* ^′^ such that $\mu _{2}|_{w}\sqsubseteq \mu ^{\prime }$, *μ* ^′^ ⊧ *R*, and *μ* ^′^(*B*) ⊆ *G*. Without loss of generality, *d* *o* *m*(*μ* ^′^) = *d* *o* *m*(*μ*_2_) ∪ *v* *a* *r* *s*(*B*). Thus, since *P* is weakly well-designed, *v* *a* *r* *s*(*B*) ∩ *d* *o* *m*(*μ*|_*u*_) ⊆ *v* *a* *r* *s*(*B* ^′^) ⊆ *d* *o* *m*(*μ*_2_). Hence, *μ* ^′^ is compatible with *μ*|_*u*_, and $\mu |_{w}\sqsubseteq \mu |_{u}\cup \mu ^{\prime }$. Moreover, since *μ* ^′^ and *μ*|_*u*_ ∪ *μ* ^′^ coincide on *v* *a* *r* *s*(*B*) and *R* is safe, we have that *μ*|_*u*_ ∪ *μ* ^′^ ⊧ *R* and (*μ*|_*u*_ ∪ *μ* ^′^)(*B*) ⊆ *G*, contradicting the assumption for *μ*. Since *μ*_2_ satisfies conditions 1–3 with respect to $\mathcal {T}(P)_{u}$, by the outer inductive hypothesis we obtain that $\mu _{2}\in [{\kern -2.3pt}[ \mathcal {T}(P)_{u} ]{\kern -2.3pt}]_{G}$, and hence $\mu \in [{\kern -2.3pt}[ \mathcal {T}(P)_{\prec u} ]{\kern -2.3pt}]_{G}\Join [{\kern -2.3pt}[ \mathcal {T}(P)_{u} ]{\kern -2.3pt}]_{G}$ (as $\mu |_{u}\in [{\kern -2.3pt}[ \mathcal {T}(P)_{\prec u} ]{\kern -2.3pt}]_{G}$ holds by the inner inductive hypothesis). The claim follows.

□

Checking whether a mapping *μ* satisfies this characterisation is feasible in coNP, and the matching lower bound follows from the coNP-hardness of evaluation of wd-patterns [33].

### **Theorem 1**

*Problem* Eval $({\mathcal {P}_{\text {wwd}}})$ *is* coNP *-complete.*

### *Proof*

The lower bound of this statement is known from [33], and the upper bound can be obtained from Lemma 3 as follows.

First we show that testing whether *μ* is a pp-solution takes polynomial time, same as computing the maximal witnessing tree $\mathcal {T}(P_{\mu })$. We just proceed from the root of the tree down along the branches until we cannot find a match *μ*(t r i p l e s(*v*)) in *G* for the basic pattern in the child *v* which satisfies the condition in the node, and then check that the variables in the resulting tree are exactly *v* *a* *r* *s*(*μ*). So, the crucial part is to check that $\mathcal {T}(P_{\mu })$ is not extendable to any of its children. But there are only linearly many children, and each check can be done in coNP. Finally, the checks for top-level filters are again polynomial. □

Pérez et al. [33] extended wd-patterns to U N I O N by considering *unions of wd-patterns*, that is, patterns of the form *P*_1_ U N I O N … U N I O N *P*_*n*_ with all $P_{i}\in \mathcal {P}_{\text {wd}}$. We denote the resulting fragment by $\mathcal {U}_{\text {wd}}$. This syntactic restriction on the use of U N I O N in $\mathcal {U}_{\text {wd}}$ is motivated by the fact that any pattern in $\mathcal {U}$ can be equivalently expressed as a union of U N I O N-free patterns [33]. We denote the fragment of all queries over patterns in $\mathcal {U}_{\text {wd}}$ by $\mathcal {S}_{\text {wd}}$. Similarly, we write $\mathcal {U}_{\text {wwd}}$ for unions of wwd-patterns and $\mathcal {S}_{\text {wwd}}$ for queries over unions of wwd-patterns.

Analogously to the well-designed case, Theorem 1 extends to fragments $\mathcal {U}_{\text {wwd}}$ and $\mathcal {S}_{\text {wwd}}$.

### **Corollary 2**

*Problem* Eval *(* ${\mathcal {U}_{\text {wwd}}}$ *)* *is* coNP *-complete, and* Eval *(* ${\mathcal {S}_{\text {wwd}}}$ *)* *is* ${{\Sigma }_{2}^{p}}$ *-complete.*

The coNP-algorithm for $\mathcal {U}_{\text {wwd}}$ is obtained simply by applying the algorithm for $\mathcal {P}_{\text {wwd}}$ to each pattern in the union. Hardness for $\mathcal {S}_{\text {wwd}}$ follows from the hardness of the well-designed case [27], while for membership we just guess the values of the existential variables and then call a coNP-oracle for $\mathcal {U}_{\text {wwd}}$ on the resulting mapping and the normalised body of the query.

Hence, the complexity of evaluation for wwd-patterns is the same as for wd-patterns. We next show that wwd-patterns are, in a certain sense, a maximal extension of wd-patterns that preserves coNP evaluation complexity (under the usual complexity-theoretic assumptions).

The definition of weakly well-designed patterns suggests two intuitive ways in which it could be relaxed. Given an occurrence *i* of an O P T-subpattern *P*_1_ O P T *P*_2_, one could allow variables in *v* *a* *r* *s*(*P*_2_) ∖ *v* *a* *r* *s*(*P*_1_) to occur in

- some subpatterns whose occurrences are not dominated by *i*, or
- constraints of some non-top-level occurrences of F I L T E R-patterns.

We next show that either relaxation immediately makes the evaluation problem ${{\Pi }_2^p}$-hard.

For the first relaxation, the arguably simplest special case would be to allow for some non-well-designed O P T-nesting of type (Opt-R). Consider the fragment $\mathcal {P}_{\text {opt-r}}$ of patterns of the form *B*_1_ O P T (*B*_2_ O P T *B*_3_), where *B*_1_, *B*_2_ and *B*_3_ are basic patterns. Intuitively, $\mathcal {P}_{\text {opt-r}}$ allows for the most simple form of non-well-designed nesting of type (Opt-R).

### **Theorem 2**

*Problem* Eval $({\mathcal {P}_{\text {opt-r}}})$ *is* ${{\Pi }_2^p}$ *-complete.*

### *Proof*

This theorem is a corollary of [38, Theorem 4] for their class ${\mathcal {E}}_{\leq 3}$, but without U N I O N. □

Now suppose we allow for some non-well-designed non-top-level filters, as suggested by the second relaxation. As we will see next, even a very restricted fragment of patterns allowing for such filters is ${{\Pi }_2^p}$-complete. This implies that the requirement that special nodes be children of the root, while it may look somewhat ad-hoc, cannot be substantially relaxed. Consider the fragment $\mathcal {P}_{\text {filter-2}}$ of patterns of the form

$$B_{1}~{{\mathsf{OPT}}}~((B_{2}~{{\mathsf{OPT}}}~B_{3})~{{\mathsf{FILTER}}}~R), $$

where *B*_1_, *B*_2_ and *B*_3_ are basic patterns such that *v* *a* *r* *s*(*B*_3_) ∩ *v* *a* *r* *s*(*B*_1_) ⊆ *v* *a* *r* *s*(*B*_2_), and *R* is a filter constraint. Intuitively, $\mathcal {P}_{\text {filter-2}}$ allows for the simplest form of “second-level” filters.

### **Theorem 3**

*Problem* Eval *(* ${\mathcal {P}_{\text {filter-2}}}$ *)* *is* ${{\Pi }_2^p}$ *-complete.*

### *Proof*

This problem allows for a reduction from a restriction of Eval(${\mathcal {P}_{\text {opt-r}}}$). Indeed, from the proof of [38, Theorem 4] it follows that it is already ${{\Pi }_2^p}$-hard to check whether *μ* ∈ [[*P*]]_*G*_ for *P* of the form *B*_1_ O P T (*B*_2_ O P T *B*_3_) with *d* *o* *m*(*μ*) = *v* *a* *r* *s*(*B*_1_) and *v* *a* *r* *s*(*B*_2_) ∖ *v* *a* *r* *s*(*B*_1_) ≠ *∅*. Let *P* and *μ* be such a pattern and such a mapping, respectively. Consider the pattern

$$\begin{array}{rcl} P^{\prime} & = & B_{1}~{{\mathsf{OPT}}}~(((B_{2} \cup B_{1})~{{\mathsf{OPT}}}~B^{\prime}_{3}) {{\mathsf{FILTER}}}~ R), \text{ where } \\ R & = & \neg bound (?x^{\prime}_{1}) \vee ((?x^{\prime}_{1} = {} ?x_{1}) \wedge {\dots} \wedge (?x^{\prime}_{n} = {} ?x_{n})), \end{array} $$

with $B^{\prime }_{3}$ a basic pattern obtained from *B*_3_ by replacing all the variables ?*x*_1_, …, ?*x*_*n*_ in (*v* *a* *r* *s*(*B*_3_) ∩ *v* *a* *r* *s*(*B*_1_)) ∖*v* *a* *r* *s*(*B*_2_) by their fresh copies $?x_{1}^{\prime }, \ldots , ?x_{n}^{\prime }$ (if no such variables exist, that is, if the original pattern is well-designed, we just set *R* to ⊤). Clearly, $P^{\prime }\in \mathcal {P}_{\text {filter-2}}$, so it suffices to show, for every *G* and *μ* with *d* *o* *m*(*μ*) = *v* *a* *r* *s*(*B*_1_), that *μ* ∈ [[*P*]]_*G*_ if and only if *μ* ∈ [[*P* ^′^]]_*G*_.

For the forward direction, suppose *d* *o* *m*(*μ*) = *v* *a* *r* *s*(*B*_1_) and *μ* ∈ [[*P*]]_*G*_. Since *v* *a* *r* *s*(*B*_2_) ∖*v* *a* *r* *s*(*B*_1_) ≠ *∅*, we must have *μ* ∈ [[*B*_1_]]_*G*_ ∖ [[*B*_2_ O P T *B*_3_]]_*G*_. Thus, *μ* ∈ [[*B*_1_]]_*G*_ and for every *μ* ^′^∈ [[*B*_2_ O P T *B*_3_]]_*G*_ we have *μ* ≁ *μ* ^′^. Since *μ* ∈ [[*B*_1_]]_*G*_, to show *μ* ∈ [[*P* ^′^]]_*G*_ it suffices to verify that *μ* is not compatible with any $\mu ^{\prime }\in [{\kern -2.3pt}[ {((B_{2} \cup B_{1})~{{\mathsf {OPT}}}~B^{\prime }_{3}) ~{{\mathsf {FILTER}}}~R}]{\kern -2.3pt}]_{G}$, for which we distinguish the following two cases.

- If $\mu ^{\prime }\in [{\kern -2.3pt}[ {B_{2}\cup B_{1}} ]{\kern -2.3pt}]_{G}\Join [{\kern -2.3pt}[ {B^{\prime }_{3}} ]{\kern -2.3pt}]_{G}$, then $\mu ^{\prime }\models (?x^{\prime }_{1} = {}?x_{1}) \wedge {\dots } \wedge (?x^{\prime }_{n} = {} ?x_{n})$. Hence, $\mu ^{\prime }|_{{\mathsf {vars}(B_{2})}\cup {\mathsf {vars}(B_{3})}}\in [{\kern -2.3pt}[ {B_{2}~{{\mathsf {OPT}}}~B_{3}} ]{\kern -2.3pt}]_{G}$. Consequently, by assumption, $\mu \not \sim \mu ^{\prime }|_{{\mathsf {vars}(B_{2})}\cup {\mathsf {vars}(B_{3})}}$, and thus *μ* ≁ *μ* ^′^.
- If $\mu ^{\prime }\in [{\kern -2.3pt}[ {B_{2}\cup B_{1}} ]{\kern -2.3pt}]_{G}\setminus [{\kern -2.3pt}[ {B^{\prime }_{3}} ]{\kern -2.3pt}]_{G}$, then $\mu ^{\prime }|_{{\mathsf {vars}(B_{2})}}\in [{\kern -2.3pt}[ {B_{2}} ]{\kern -2.3pt}]_{G}\setminus [{\kern -2.3pt}[ {B^{\prime }_{3}} ]{\kern -2.3pt}]_{G}=[{\kern -2.3pt}[ {B_{2}} ]{\kern -2.3pt}]_{G}\setminus [{\kern -2.3pt}[ {B_{3}} ]{\kern -2.3pt}]_{G}\subseteq [{\kern -2.3pt}[ {B_{2}}~{{\mathsf {OPT}}}~B_{3}]{\kern -2.3pt}]_{G}$. Therefore, by assumption, $\mu \not \sim \mu ^{\prime }|_{{\mathsf {vars}(B_{2})}}$, and hence *μ* ≁ *μ* ^′^.

For the backward direction, suppose *d* *o* *m*(*μ*) = *v* *a* *r* *s*(*B*_1_) and $\mu \in [{\kern -2.3pt}[ P^{\prime } ]{\kern -2.3pt}]_{G}$. Again, since *v* *a* *r* *s*(*B*_2_) ∖*v* *a* *r* *s*(*B*_1_) ≠ *∅*, we have $\mu \in [{\kern -2.3pt}[ {B_{1}} ]{\kern -2.3pt}]_{G}\setminus [{\kern -2.3pt}[ ((B_{2} \cup B_{1})~{{\mathsf {OPT}}}~B^{\prime }_{3})$ F I L T E R *R*]]_*G*_. Thus, *μ* ∈ [[*B*_1_]]_*G*_ and *μ* ≁ *μ* ^′^ for every $\mu ^{\prime }\in [{\kern -2.3pt}[ ((B_{2} \cup B_{1})~{{\mathsf {OPT}}}~B^{\prime }_{3})$ F I L T E R *R*]]_*G*_. Since *μ* ∈ [[*B*_1_]]_*G*_, it follows that $[{\kern -2.3pt}[ {((B_{2} \cup B_{1})~{{\mathsf {OPT}}}~B^{\prime }_{3})~{{\mathsf {FILTER}}}~R} ]{\kern -2.3pt}]_{G} =\emptyset $. To show *μ* ∈ [[*P*]]_*G*_ it suffices to verify that *μ* is not compatible with any $\mu ^{\prime }\in [{\kern -2.3pt}[ {B_{2}\,{{\mathsf {OPT}}}\, B_{3}} ]{\kern -2.3pt}]_{G}$. Assume for the sake of contradiction that this is not the case and there is a compatible *μ* ^′^. We distinguish the following two cases.

- Suppose $\mu ^{\prime }\in [{\kern -2.3pt}[ {B_{2}} ]{\kern -2.3pt}]_{G}\Join [{\kern -2.3pt}[ {B_{3}} ]{\kern -2.3pt}]_{G}$. Then there is some *μ* ^″^ such that $\mu \cup \mu ^{\prime }\cup \mu ^{\prime \prime }\in [{\kern -2.3pt}[ {B_{1}} ]{\kern -2.3pt}]_{G}\Join [{\kern -2.3pt}[ {B_{2}} ]{\kern -2.3pt}]_{G}\Join [{\kern -2.3pt}[ {B^{\prime }_{3}} ]{\kern -2.3pt}]_{G} $ and $\mu \cup \mu ^{\prime }\cup \mu ^{\prime \prime }\models (?x^{\prime }_{1} = {} ?x_{1}) \wedge {\dots } \wedge (?x^{\prime }_{n} = {} ?x_{n})$. Thus, $\mu \cup \mu ^{\prime }\cup \mu ^{\prime \prime }\in [{\kern -2.3pt}[ {((B_{2} \cup B_{1})~{{\mathsf {OPT}}}~ B^{\prime }_{3})~{{\mathsf {FILTER}}}~R} ]{\kern -2.3pt}]_{G} $, which is a contradiction.
- Suppose $\mu ^{\prime }\in [{\kern -2.3pt}[ {B_{2}} ]{\kern -2.3pt}]_{G}\setminus [{\kern -2.3pt}[ {B_{3}} ]{\kern -2.3pt}]_{G}=[{\kern -2.3pt}[ {B_{2}} ]{\kern -2.3pt}]_{G}\setminus [{\kern -2.3pt}[ {B^{\prime }_{3}} ]{\kern -2.3pt}]_{G} $. Then $\mu \cup \mu ^{\prime }\in [{\kern -2.3pt}[ {B_{1}} ]{\kern -2.3pt}]_{G}\cup B_{2}\setminus [{\kern -2.3pt}[ {B^{\prime }_{3}} ]{\kern -2.3pt}]_{G} $ and $\mu \cup \mu ^{\prime }\models \neg bound(?x^{\prime }_{1})$, and hence $\mu \cup \mu ^{\prime }\in [{\kern -2.3pt}[ {((B_{2} \cup B_{1})~{{\mathsf {OPT}}}~B^{\prime }_{3})~\mathsf {FILTER}~R} ]{\kern -2.3pt}]_{G} $, which is again a contradiction.

□

Theorems 2 and 3 suggest that $\mathcal {P}_{\text {wwd}}$ is a maximal fragment of $\mathcal {P}$ that does not impose structural restrictions on basic patterns or filter constraints and has a coNP evaluation algorithm (assuming $\text {\textsc {coNP}} \neq {{\Pi }_2^p}$). Hence, going beyond wwd-patterns while preserving good computational properties requires more refined restrictions, possibly in the spirit of [27, Section 4].

## Expressivity of wwd-Patterns and Their Extensions

In this section, we analyse the expressive power of our fragments.

### **Definition 9**

A language $\mathcal {L}_{1}$ is *strictly less expressive* than a language $\mathcal {L}_{2}$ (written $\mathcal {L}_{1}<\mathcal {L}_{2}$) if for every query *Q*_1_ in $\mathcal {L}_{1}$ there is a query *Q*_2_ in $\mathcal {L}_{2}$ such that *Q*_1_ ≡ *Q*_2_, and there is a query *Q*_2_ in $\mathcal {L}_{2}$ such that *Q*_1_ ≢ *Q*_2_ for every query *Q*_1_ in $\mathcal {L}_{1}$.

We begin with UNION-free patterns, establishing that $\mathcal {P}_{\text {wd}}<\mathcal {P}_{\text {wwd}}<\mathcal {P}$, and then proceed to unions, showing that $\mathcal {U}_{\text {wd}}<\mathcal {U}_{\text {wwd}}<\mathcal {U}$, and queries, showing that $\mathcal {S}_{\text {wd}}<\mathcal {S}_{\text {wwd}}<\mathcal {S}$.

Following [5, 33], a set of mappings Ω_1_ is *subsumed* by a set of mappings Ω_2_ (written ${\Omega }_{1} \sqsubseteq {\Omega }_{2}$) if for every *μ*_1_ ∈ Ω_1_ there exists a mapping *μ*_2_ ∈ Ω_2_ such that $\mu _{1} \sqsubseteq \mu _{2}$. A query *Q* is *weakly monotone* if $[{\kern -2.3pt}[ Q ]{\kern -2.3pt}]_{G_{1}} \sqsubseteq [{\kern -2.3pt}[ Q ]{\kern -2.3pt}]_{G_{2}}$ for any two graphs *G*_1_ and *G*_2_ with *G*_1_ ⊆ *G*_2_, and a fragment $\mathcal {L}$ is *weakly monotone* if it contains only weakly monotone queries. Arenas and Pérez [5] showed that, unlike $\mathcal {P}$, the fragment $\mathcal {P}_{\text {wd}}$ is weakly monotone, and hence $\mathcal {P}_{\text {wd}}<\mathcal {P}$.

### *Example 4*

(Pérez et al. [33]) Consider the non-well-designed pattern

$$\begin{array}{*{20}l} P = {(?x, a, 1)}~{{\mathsf{OPT}}}~({(?y, a, 2)}~{{\mathsf{OPT}}}~{(?x, a, 3)}) \end{array} $$

as well as graphs *G*_1_ = {(1, *a*, 1), (2, *a*, 2)} and *G*_2_ = *G*_1_ ∪ {(3, *a*, 3)}. Then *μ*_1_ = {?*x* ↦ 1, ?*y* ↦ 2} is the only mapping in $[{\kern -2.3pt}[ P ]{\kern -2.3pt}]_{G_{1}}$ while *μ*_2_ = {?*x*↦1} is the only mapping in $[{\kern -2.3pt}[ P ]{\kern -2.3pt}]_{G_{2}}$. Hence $[{\kern -2.3pt}[ P ]{\kern -2.3pt}]_{G_{1}}\not \sqsubseteq [{\kern -2.3pt}[ P ]{\kern -2.3pt}]_{G_{2}}$, meaning that *P* is not weakly monotone.

Analogously, we show that $\mathcal {P}_{\text {wd}}<\mathcal {P}_{\text {wwd}}$ by observing that $\mathcal {P}_{\text {wwd}}$ is not weakly monotone.

### **Proposition 7**

*Fragment* $\mathcal {P}_{\text {wwd}}$ *is not weakly monotone.*

### *Proof*

Consider a wwd-pattern

$$P=({(?x, a, 1)}~{{\mathsf{OPT}}}~{(?y, a, 2)})~{{\mathsf{OPT}}}~{(?y, a, 3)}, $$

as well as graphs *G*_1_ = {(1, *a*,1),(3, *a*,3)} and *G*_2_ = *G*_1_ ∪{(2, *a*,2)}. Then $[{\kern -2.3pt}[ P ]{\kern -2.3pt}]_{{G_{1}}}=\{\{?x\mapsto 1,?y\mapsto 3\}\}\not \sqsubseteq \{\{?x\mapsto 1,?y\mapsto 2\}\}=[{\kern -2.3pt}[ P ]{\kern -2.3pt}]_{{G_{2}}}$. □

An alternative proof of $\mathcal {P}_{\text {wd}}<\mathcal {P}_{\text {wwd}}$ can be obtained by adapting Theorem 3.5 in [6], which exhibits a weakly well-designed, weakly monotone pattern that is not equivalent to any well-designed pattern.

To distinguish $\mathcal {P}_{\text {wwd}}$ from $\mathcal {P}$ we need a different property.

### **Definition 10**

A query *Q* is *non-reducing* if for any two graphs *G*_1_, *G*_2_ such that *G*_1_ ⊆ *G*_2_ and any mapping $\mu _{1} \in [{\kern -2.3pt}[ Q ]{\kern -2.3pt}]_{G_{1}}$ there is no $\mu _{2} \in [{\kern -2.3pt}[ Q ]{\kern -2.3pt}]_{G_{2}}$ such that $\mu _{2} \sqsubset \mu _{1}$ (i.e., $\mu _{2} \sqsubseteq \mu _{1}$ and *μ*_2_ ≠ *μ*_1_). A fragment is *non-reducing* if it contains only non-reducing queries.

Intuitively, for a non-reducing query extending a graph cannot result in a previously bound answer variable becoming unbound. All weakly monotone queries are non-reducing but not vice versa. Moreover, all wwd-patterns are non-reducing.

### **Proposition 8**

*Fragment* $\mathcal {P}_{\text {wwd}}$ *is non-reducing.*

### *Proof*

Let $P\in \mathcal {P}_{\text {wwd}}$ and let *G*_1_, *G*_2_ be two graphs such that *G*_1_ ⊆ *G*_2_. We show that $\mu _{2}\not \sqsubset \mu _{1}$ for any $\mu _{1}\in [{\kern -2.3pt}[ P ]{\kern -2.3pt}]_{{G_{1}}}$ and $\mu _{2}\in [{\kern -2.3pt}[ P ]{\kern -2.3pt}]_{{G_{2}}}$ by induction on the structure of *P*, proving, in parallel, that if all filters in *P* are over basic patterns, then for every mapping $\mu _{1}\in [{\kern -2.3pt}[ P ]{\kern -2.3pt}]_{{G_{1}}}$ there is a mapping $\mu _{2}\in [{\kern -2.3pt}[ P ]{\kern -2.3pt}]_{{G_{2}}}$ such that *μ*_1_|_*vars*(*v*)_ = *μ*_2_|_*vars*(*v*)_ for *v* the root of $\mathcal {T}(P)$.

For the base case, suppose *P* = *B* F I L T E R *R* for some basic pattern *B* and filter constraint *R*. Then, *P* is *monotone* in the sense of [5], that is, satisfies $[{\kern -2.3pt}[ P ]{\kern -2.3pt}]_{{G_{1}}}\subseteq [{\kern -2.3pt}[ P ]{\kern -2.3pt}]_{{G_{2}}}$. Moreover, *P* contains no OPT, and hence every two distinct mappings in $[{\kern -2.3pt}[ P ]{\kern -2.3pt}]_{G_{2}}$ have the same domain and are thus incompatible. These facts imply both claims.

For the inductive step, suppose first that *P* = *P*_1_ O P T *P*_2_ and both claims hold for *P*_1_ and *P*_2_. Let $\mu _{1}\in [{\kern -2.3pt}[ P ]{\kern -2.3pt}]_{G_{1}}$. We first prove that $\mu _{2}\not \sqsubset \mu _{1}$ for any $\mu _{2}\in [{\kern -2.3pt}[ P ]{\kern -2.3pt}]_{{G_{2}}}$. We distinguish two cases.

- Let $\mu _{1}={\mu _{1}^{1}}{\cup \mu _{1}^{2}}$ where ${\mu _{1}^{i}}\in [{\kern -2.3pt}[ P_{i} ]{\kern -2.3pt}]_{G_{1}}$. Assume for contradiction that $\mu _{2}\sqsubset \mu _{1}$ for some $\mu _{2}\in [{\kern -2.3pt}[ P ]{\kern -2.3pt}]_{{G_{2}}}$. We begin by showing that *μ*_2_ must be of the form ${\mu _{2}^{1}}{\cup \mu _{2}^{2}}$ where ${\mu _{2}^{i}}\in [{\kern -2.3pt}[ P_{i} ]{\kern -2.3pt}]_{G_{2}}$, for which it suffices to show that $\mu _{2}|_{{\mathsf {vars}(P_{1})}}$ is compatible with some mapping in $[{\kern -2.3pt}[ P_{2} ]{\kern -2.3pt}]_{G_{2}}$. On the one hand, since $\mu _{2}\sqsubset \mu _{1}$, ${\mu _{1}^{2}}$ is compatible with $\mu _{2}|_{{\mathsf {vars}(P_{1})}}$. On the other hand, since all filters in *P*_2_ are over basic patterns, the inductive hypothesis tells us that $[{\kern -2.3pt}[ P_{2} ]{\kern -2.3pt}]_{G_{2}}$ contains a mapping *μ* ^′^ that coincides with ${\mu _{1}^{2}}$ on the set of variables *X* in the root of $\mathcal {T}(P_{2})$; moreover, since *P* is weakly well-designed, *d* *o* *m*(*μ* ^′^) ∩ *v* *a* *r* *s*(*P*_1_) ⊆ *X*, and hence *μ* ^′^ is compatible with $\mu _{2}|_{{\mathsf {vars}(P_{1})}}$. Thus, $\mu _{2}={\mu _{2}^{1}}{\cup \mu _{2}^{2}}$ where ${\mu _{2}^{i}}\in [{\kern -2.3pt}[ P_{i} ]{\kern -2.3pt}]_{G_{2}}$. Then, however, we must have that ${\mu _{2}^{1}}{\sqsubset \mu _{1}^{1}}$ or ${\mu _{2}^{2}}{\sqsubset \mu _{1}^{2}}$, contradicting the inductive hypothesis for *P*_1_ or *P*_2_, respectively.
- Let $\mu _{1}\in [{\kern -2.3pt}[ P_{1} ]{\kern -2.3pt}]_{G_{1}}\setminus [{\kern -2.3pt}[ P_{2} ]{\kern -2.3pt}]_{G_{1}}$, and let *μ*_2_ be an arbitrary mapping in $[{\kern -2.3pt}[ P ]{\kern -2.3pt}]_{{G_{2}}}$. Then *μ*_2_ extends some $\mu ^{\prime }\in [{\kern -2.3pt}[ P_{1} ]{\kern -2.3pt}]_{G_{2}}$. By the inductive hypothesis for claim 2, we have that $\mu ^{\prime }\not \sqsubset \mu _{1}$, and hence $\mu _{2}\not \sqsubset \mu _{1}$.

Suppose now that all filters in *P* are over basic patterns. We need to prove that there is $\mu _{2}\in [{\kern -2.3pt}[ P ]{\kern -2.3pt}]_{{G_{2}}}$ such that *μ*_1_|_*vars*(*v*)_ = *μ*_2_|_*vars*(*v*)_. We know that *μ*_1_ extends some $\mu ^{\prime }_{1}\in [{\kern -2.3pt}[ P_{1} ]{\kern -2.3pt}]_{G_{1}}$. Thus, by the inductive hypothesis, there is some $\mu ^{\prime }_{2}\in [{\kern -2.3pt}[ P_{1} ]{\kern -2.3pt}]_{G_{2}}$ that coincides with $\mu ^{\prime }_{1}$ on the variables in the root of $\mathcal {T}(P_{1})$. The claim follows since $\mu ^{\prime }_{2}$ can be extended to a mapping *μ*_2_ for *P* that coincides with $\mu ^{\prime }_{2}$ on the variables in the root of $\mathcal {T}(P_{1})$, and, by construction, the root of $\mathcal {T}(P_{1})$ and the root of $\mathcal {T}(P)$ have the same label.

Consider now the inductive step for the case when *P* = *P*_1_ F I L T E R *R*. Since *P*_1_ is not a basic pattern, we only need to show that $\mu _{2}\not \sqsubset \mu _{1}$ for any $\mu _{1}\in [{\kern -2.3pt}[ P ]{\kern -2.3pt}]_{{G_{1}}}$ and $\mu _{2}\in [{\kern -2.3pt}[ P ]{\kern -2.3pt}]_{{G_{2}}}$. This holds by the inductive hypothesis, because $\mu _{1}\in [{\kern -2.3pt}[ P_{1} ]{\kern -2.3pt}]_{G_{1}}$ and $\mu _{2}\in [{\kern -2.3pt}[ P_{1} ]{\kern -2.3pt}]_{G_{2}}$ for any such *μ*_1_ and *μ*_2_. □

In contrast to Proposition 8, patterns in $\mathcal {P}$ do not generally satisfy non-reducibility. For instance, consider again pattern *P*, graphs *G*_1_, *G*_2_, and mappings *μ*_1_, *μ*_2_ from Example 4. Pattern *P* is not non-reducing since $\mu _{1}\in [{\kern -2.3pt}[ P ]{\kern -2.3pt}]_{G_{1}}$ and $\mu _{2}\in [{\kern -2.3pt}[ P ]{\kern -2.3pt}]_{G_{2}}$ but $\mu _{2}\sqsubset \mu _{1}$. Therefore, we have the following theorem.

### **Theorem 4**

*It holds that* $\mathcal {P}_{\text {wd}}<\mathcal {P}_{\text {wwd}}<\mathcal {P}$ *.*

We next compare $\mathcal {U}_{\text {wwd}}$ to $\mathcal {U}_{\text {wd}}$ and $\mathcal {U}$, as well as $\mathcal {S}_{\text {wwd}}$ to $\mathcal {S}_{\text {wd}}$ and $\mathcal {S}$ (note that neither *UNION* nor projection via S E L E C Tcan be expressed by means of the other operators [40], so adding either construct makes each fragment strictly more expressive). It is easily seen that $\mathcal {U}_{\text {wd}}$ and $\mathcal {S}_{\text {wd}}$ inherit weak monotonicity from $\mathcal {P}_{\text {wd}}$ [27, 33], and hence $\mathcal {U}_{\text {wd}}<\mathcal {U}_{\text {wwd}}$ and $\mathcal {S}_{\text {wd}}<\mathcal {S}_{\text {wwd}}$. Non-reducibility, however, does not propagate to unions.

### *Example 5*

Consider the following $\mathcal {U}_{\text {wd}}$-pattern with *G*_1_, *G*_2_ and *μ*_1_, *μ*_2_ from Example 4:

$$\begin{array}{*{20}l} P = ({(?x, a, 1)}~{{\mathsf{OPT}}}~{(?y, a, 2)})~{{\mathsf{UNION}}}~{(?x, a, 1)}. \end{array} $$

We have $\mu _{1}\in [{\kern -2.3pt}[ P ]{\kern -2.3pt}]_{G_{1}}$ and $\mu _{2}\in [{\kern -2.3pt}[ P ]{\kern -2.3pt}]_{G_{2}}$ but $\mu _{2}\sqsubset \mu _{1}$, which is due to the fact that *μ*_2_ is already contained in $[{\kern -2.3pt}[ P ]{\kern -2.3pt}]_{G_{1}}$ along with *μ*_1_. This is only possible in the presence of UNION since all mappings in the evaluation of a UNION-free pattern are mutually non-subsuming (see Lemma 2).

Thus, to account for UNION, we introduce the following, more delicate property.

### **Definition 11**

A query *Q* is *extension-witnessing* (*e-witnessing*) if for any two graphs *G*_1_ ⊆ *G*_2_ and mapping $\mu \!\in \![{\kern -2.3pt}[ Q ]{\kern -2.3pt}]_{G_{2}}$ such that $\mu \!\notin \![{\kern -2.3pt}[ Q ]{\kern -2.3pt}]_{G_{1}}\!$ there is a triple *t* in *Q* such that *v* *a* *r* *s*(*t*) ⊆ *d* *o* *m*(*μ*) and *μ*(*t*) ∈ *G*_2_ ∖ *G*_1_. A fragment is *e-witnessing* if so are all of its queries.

Informally, a query *Q* is e-witnessing if whenever an extension of a graph leads to a new answer, this answer is justified by a triple pattern in *Q* which maps to the extension. Unions of wwd-patterns can be shown e-witnessing.

### **Proposition 9**

*Fragment* $\mathcal {U}_{\text {wwd}}$ *is e-witnessing.*

### *Proof*

Let $P\in \mathcal {U}_{\text {wwd}}$ and let *G*_1_, *G*_2_ be graphs such that *G*_1_ ⊆ *G*_2_. Let *μ* be a mapping in $[{\kern -2.3pt}[ P ]{\kern -2.3pt}]_{G_{2}}$ but not in $[{\kern -2.3pt}[ P ]{\kern -2.3pt}]_{{G_{1}}}$. We show that there is some *t* ∈ t r i p l e s(*P*) such that *μ*(*t*) ∈ *G*_2_ ∖ *G*_1_.

Since *P* is a union of wwd-patterns, there is some wwd-pattern *P* ^′^ in the union such that $\mu \in [{\kern -2.3pt}[ P^{\prime } ]{\kern -2.3pt}]_{G_{2}}$. It suffices to show $\mu (\mathsf {triples}(P^{\prime }_{\mu }))\cap (G_{2}\setminus G_{1})\ne \emptyset $, where $P^{\prime }_{\mu }$ is the pattern corresponding to the maximal r-subtree of *P* witnessing *μ* in *G*_2_ (i.e., the part of *P* in the image of *μ*, see Definition 8). We know that $\mu (\mathsf {triples}(P^{\prime }_{\mu })) \subseteq G_{2}$. Assume, for contradiction, that $\mu (\mathsf {triples}(P^{\prime }_{\mu }))\subseteq G_{1}$. Then *μ* is a pp-solution to *P* ^′^ over *G*_1_. We next show that *μ* is a real solution to *P* ^′^ over *G*_1_. By Lemma 3, it suffices to show that (a) for any child *u* of $\mathcal {T}(P^{\prime }_{\mu })$ labelled with (*B*, *R*), there is no mapping *μ* ^′^ such that $\mu |_{u}\sqsubseteq \mu ^{\prime }$, *μ* ^′^ ⊧ *R*, and *μ* ^′^(*B*) ⊆ *G*_1_, and (b) *μ*|_*s*_ ⊧ *R* for any special node *s* in $\mathcal {T}(P^{\prime })$ labelled with *R*. Claim (a) holds since $\mu \in [{\kern -2.3pt}[ P^{\prime } ]{\kern -2.3pt}]_{G_{2}}$ and *G*_1_ ⊆ *G*_2_ while (b) holds since $\mu \in [{\kern -2.3pt}[ P^{\prime } ]{\kern -2.3pt}]_{G_{2}}$ and the claim does not depend on the graph over which the evaluation is computed. Consequently, $\mu \in [{\kern -2.3pt}[ P^{\prime } ]{\kern -2.3pt}]_{G_{1}}$, and hence $\mu \in [{\kern -2.3pt}[ P ]{\kern -2.3pt}]_{{G_{1}}}$, in contradiction to the assumption. □

On the other hand, $\mathcal {U}$ is not e-witnessing, as can be seen on the pattern and graphs in Example 4. Hence, we obtain the following theorem.

### **Theorem 5**

*It holds that* $\mathcal {U}_{\text {wd}}<\mathcal {U}_{\text {wwd}}<\mathcal {U}$ *.*

Next we move to the fragments that allow for projection. As already mentioned, we have $\mathcal {S}_{\text {wd}}<\mathcal {S}_{\text {wwd}}$ since $\mathcal {S}_{\text {wd}}$ is weakly monotone while $\mathcal {S}_{\text {wwd}}$ is not. However, $\mathcal {S}_{\text {wwd}}$ is not e-witnessing, so we cannot apply the technique of Theorem 5 to establish $\mathcal {S}_{\text {wwd}}<\mathcal {S}$; instead, we make use of the following lemma.

### **Lemma 4**

*Let Q be a query in* $\mathcal {S}_{\text {wwd}}$ *and G be a graph. For every graph* *G*_1_ *with* *G* ⊆ *G*_1_ *and every* $\mu \in [{\kern -2.3pt}[ Q ]{\kern -2.3pt}]_{{G_{1}}}$ *,* *there is a graph* *G*_2_ *with* *G* ⊆ *G*_2_ *such that* $\mu \in [{\kern -2.3pt}[ Q ]{\kern -2.3pt}]_{{G_{2}}}$ *and* |*G*_2_| ≤ |*G*| + |t r i p l e s(*Q*)|*.*

### *Proof*

Let *Q* = S E L E C T *X* *W* *H* *E* *R* *E* *P*, for *P* a union of wwd-patterns, and let *G*, *G*_1_ and *μ* be as required. Then there is a wwd-pattern *P* ^′^ in the union *P* such that $\mu ^{\prime }\in [{\kern -2.3pt}[ P^{\prime } ]{\kern -2.3pt}]_{G_{1}}$ for some *μ* ^′^ with *μ* ^′^|_*X*_ = *μ*. Let $G_{2}=G\cup \mu ^{\prime }(\mathsf {triples}(P^{\prime }_{\mu ^{\prime }}))$. Clearly, |*G*_2_| ≤ |*G*| + |t r i p l e s(*Q*)|, so it suffices to show that $\mu ^{\prime }\in [{\kern -2.3pt}[ P^{\prime } ]{\kern -2.3pt}]_{G_{2}}$.

By construction, *μ* ^′^ is a pp-solution to *P* ^′^ over *G*_2_. Moreover, since *μ* ^′^ is a solution to *P* ^′^ over *G*_1_, we have that *μ*|_*s*_ ⊧ *R* for every special node *s* in $\mathcal {T}(P^{\prime })$ labelled with *R*. Finally, suppose for contradiction that there is a child *v* of $\mathcal {T}(P^{\prime }_{\mu ^{\prime }})$ labelled with (*B*, *R*) and a mapping *μ* ^″^ such that $\mu ^{\prime }|_{v}\sqsubseteq \mu ^{\prime \prime }$, *μ* ^″^ ⊧ *R*, and *μ* ^″^(*B*) ⊆ *G*_2_. However, since *G*_2_ ⊆ *G*_1_, we then have *μ* ^″^(*B*) ⊆ *G*_1_, which contradicts the fact that $\mu ^{\prime }\in [{\kern -2.3pt}[ P^{\prime } ]{\kern -2.3pt}]_{G_{1}}$. □

This lemma is the base of the last result of the section.

### **Theorem 6**

*It holds that* $\mathcal {S}_{\text {wd}}<\mathcal {S}_{\text {wwd}}<\mathcal {S}$ *.*

### *Proof*

As observed before, the inclusion $\mathcal {S}_{\text {wd}}<\mathcal {S}_{\text {wwd}}$ holds since $\mathcal {S}_{\text {wd}}$ is weakly monotone [27, 33] and $\mathcal {S}_{\text {wwd}}$ is not.

As for the second inclusion, consider the family of graphs

$$G_{n}=\{{(a, a, a)} ,{({d_{1}}, b, b)} ,\dots,{({d_{n}}, b, b)} ,{({d_{1}}, c, c)} \}, $$

for pairwise distinct IRIs *a*, *b*, *c*, *d*_1_, …, *d*_*n*_, and the query

$$Q=\mathsf{SELECT} \; \{?x, ?y\} \; \mathsf{WHERE} \; {(?x, a, a)}~{\mathsf{DIFF}}~({(?y, b, b)}~{\mathsf{DIFF}}~{(?y, c, c)}). $$

By equivalence (5) in Section 3, the operator D I F F can be expressed via O P T, A N D and F I L T E R, so we can assume that $Q \in \mathcal {S}$. On the other hand, it is easily seen that $Q\notin \mathcal {S}_{\text {wwd}}$. The mapping *μ* = {?*x* ↦ *a*} is an answer to *Q* over *G*_1_ but not an answer over any *G*_*n*_ with *n* ≥ 2. Moreover, it is easily seen that any extension *G* of *G*_*n*_ such that *μ* ∈ [[*Q*]]_*G*_ requires the addition of at least *n* − 1 triples, namely {(*d*_2_, *c*, *c*), …, (*d*_*n*_, *c*, *c*)}. Consequently, *μ* ∈ [[*Q*]]_*G*_ implies |*G*| ≥ |*G*_1_| + *n* − 1.

Suppose for contradiction there is a query *Q* ^′^ in $\mathcal {S}_{\text {wwd}}$ such that *Q* ^′^ ≡ *Q*. Let *n* = |t r i p l e s(*Q* ^′^)| + 2. Then, by Lemma 4, *μ* ∈ [[*Q* ^′^]]_*G*_ for some *G* with |*G*| ≤ |*G*_*n*_| + |t r i p l e s(*Q* ^′^)| = |*G*_*n*_| + *n* − 2, which contradicts the above observation for *Q*. □

## Static Analysis of wwd-Patterns

In this section, we look at the general static analysis problems of query equivalence and containment. Formally, equivalence for a language $\mathcal {L}$ is defined as follows. 
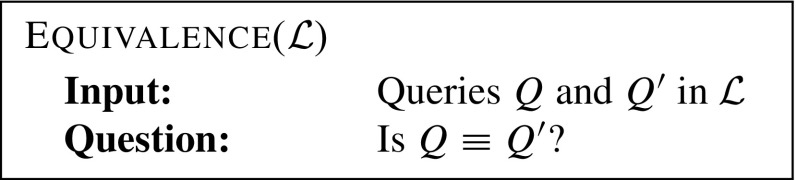


This problem is commonly generalised to $\textsc {Containment}({\mathcal {L}})$, in which one checks whether *Q* is *contained* in *Q* ^′^, written *Q* ⊆ *Q* ^′^, that is, whether [[*Q*]]_*G*_ ⊆ [[*Q* ^′^]]_*G*_ holds for every graph *G*. We have *Q* ≡ *Q* ^′^ if and only if *Q* and *Q* ^′^ contain each other.

These problems have been studied for F I L T E R-free wd-patterns in [27, 35], establishing NP-completeness of equivalence and containment. Moreover, both problems are ${{\Pi }_{2}^{p}}$-complete for unions of F I L T E R-free wd-patterns, and undecidable for fragments with projection. Finally, from the results in [41] it follows that containment is undecidable for $\mathcal {U}$. On the other hand, nothing seems to be known so far for well-designed patterns with F I L T E R.

We next show that equivalence and containment are both ${{\Pi }_2^p}$-complete for $\mathcal {P}_{\text {wwd}}$ and $\mathcal {U}_{\text {wwd}}$ (whereas they are undecidable for $\mathcal {S}_{\text {wwd}}$ by the results in [35]). As the following lemma shows, the upper bound for containment follows from a small counterexample property: if *P* ⊈ *P* ^′^ for some *P* and *P* ^′^ from $\mathcal {U}_{\text {wwd}}$, then there is a witnessing mapping and graph of size *O*(|*P*| + |*P* ^′^|). Given this property, a ${{\Pi }_2^p}$ algorithm for containment is straightforward—we guess a mapping *μ* and a graph *G* of linear size, check that *μ* ∉ [[*P* ^′^]]_*G*_, and then call a coNP oracle for checking *μ* ∈ [[*P*]]_*G*_. As a corollary, Equivalence(${\mathcal {U}_{\text {wwd}}}$) is also in ${{\Pi }_{2}^{p}}$.

### **Lemma 5**

*Let P and* *P* ^′^ *be two patterns from* $\mathcal {U}_{\text {wwd}}$ *.* *If* *P* ⊈ *P* ^′^ *then there exists a mapping* *μ* *and a graph G of size* *O*(|t r i p l e s(*P*)| + |t r i p l e s(*P* ^′^)|) *such that* *μ* ∈ [[*P*]]_*G*_ *but* *μ* ∉ [[*P* ^′^]]_*G*_ *.*

### *Proof*

Without loss of generality, let us assume that

$$\begin{array}{lclllllll} P & = & P_{1} & {{\mathsf{UNION}}} & P_{2} & {{\mathsf{UNION}}} & {\dots} & {{\mathsf{UNION}}} & P_{n}, \\ P^{\prime} & = & P^{\prime}_{1} & {{\mathsf{UNION}}} & P^{\prime}_{2} & {{\mathsf{UNION}}} & {\dots} & {{\mathsf{UNION}}} & P^{\prime}_{m}, \end{array} $$

where all *P*_*i*_ and $P^{\prime }_{j}$ are wwd-patterns in O F-normal form.

Since *P* ⊈ *P* ^′^, there exists a graph *G* ^′^, a mapping *μ*, and a pattern *P*_*i*_, 1 ≤ *i* ≤ *n*, such that $\mu \in [{\kern -2.3pt}[ P_{i} ]{\kern -2.3pt}]_{G^{\prime }}$, but $\mu \notin [{\kern -2.3pt}[ P^{\prime }_{j} ]{\kern -2.3pt}]_{G^{\prime }}$ for every $P^{\prime }_{j}$. Hence, *μ* is a pp-solution to *P*_*i*_ over *G* ^′^ with corresponding r-subtree $\mathcal {T}((P_{i})_{\mu })$ of the CPT $\mathcal {T}(P_{i})$. Let *G*_0_ = *μ*(t r i p l e s((*P*_*i*_)_*μ*_)). By construction, we have that *G*_0_ ⊆ *G* ^′^ and |*G*_0_| ≤ |t r i p l e s((*P*_*i*_)_*μ*_)| ≤ |t r i p l e s(*P*_*i*_)| ≤ |t r i p l e s(*P*)|. Moreover, $\mu \in [{\kern -2.3pt}[ P_{i} ]{\kern -2.3pt}]_{G_{0}}$, because all the matches and constraints, including the ones on the top-level, stay unchanged. In fact, $\mu \in [{\kern -2.3pt}[ P_{i} ]{\kern -2.3pt}]_{G^{\prime \prime }}$ for any *G* ^″^ such that *G*_0_ ⊆ *G* ^″^⊆ *G* ^′^.

If $\mu \notin [{\kern -2.3pt}[ P^{\prime }_{j} ]{\kern -2.3pt}]_{G_{0}}$ for every *j*, then *G*_0_ satisfies all the properties required from *G*. Otherwise, there exists $P^{\prime }_{j}$ among $P^{\prime }_{1}, \ldots , P^{\prime }_{m}$ such that $\mu \in [{\kern -2.3pt}[ P^{\prime }_{j} ]{\kern -2.3pt}]_{G_{0}}$. Since *G*_0_ ⊆ *G* ^′^, *μ* is a pp-solution to $P^{\prime }_{j}$ over *G* ^′^. Consider the corresponding pattern $(P^{\prime }_{j})_{\mu }$ (i.e., the maximal pattern witnessing *μ* in *G* ^′^ obtained from $P^{\prime }_{j}$ by dropping the right arguments of some O P T operators), the r-subtree $\mathcal {T}((P^{\prime }_{j})_{\mu })$ of the CPT $\mathcal {T}(P^{\prime }_{j})$, and the “image” $\mu (\mathsf {triples}((P^{\prime }_{j})_{\mu }))$. Note that we may have $\mu (\mathsf {triples}((P^{\prime }_{j})_{\mu })) \subseteq G_{0}$ or not: the latter is possible because the maximal r-subtree of $\mathcal {T}(P^{\prime }_{j})$ witnessing *μ* in *G*_0_ may be different from $\mathcal {T}((P^{\prime }_{j})_{\mu })$, which is maximal in *G* ^′^. Let $G^{\prime }_{1} = G_{0} \cup \mu (\mathsf {triples}((P^{\prime }_{j})_{\mu }))$. We define *G*_1_ depending on whether $\mu \in [{\kern -2.3pt}[ P^{\prime }_{j} ]{\kern -2.3pt}]_{G^{\prime }_{1}}$ or not. If $\mu \notin [{\kern -2.3pt}[ P^{\prime }_{j} ]{\kern -2.3pt}]_{G^{\prime }_{1}}$, then let $G_{1} = G^{\prime }_{1}$. Otherwise, since $\mu \notin [{\kern -2.3pt}[ P^{\prime }_{j} ]{\kern -2.3pt}]_{G^{\prime }}$ by assumption, there exists a child *v* of $\mathcal {T}((P^{\prime }_{j})_{\mu })$ and a mapping *μ*_0_ such that $\mu |_{v}\sqsubset \mu _{0}$ and *μ*_0_(t r i p l e s(*v*)) ⊆ *G* ^′^. Then the graph $G_{1} = G^{\prime }_{1} \cup \mu _{0}(\mathsf {triples}(v))$ is such that $\mu \notin [{\kern -2.3pt}[ P^{\prime }_{j} ]{\kern -2.3pt}]_{G_{1}}$. In either case, $\mu \in [{\kern -2.3pt}[ P_{i} ]{\kern -2.3pt}]_{G_{1}}$ because *G*_0_ ⊆ *G*_1_ ⊆ *G* ^′^. Moreover, we have $\mu \notin [{\kern -2.3pt}[ P^{\prime }_{j} ]{\kern -2.3pt}]_{G^{\prime \prime }}$ for every *G* ^″^ such that *G*_1_ ⊆ *G* ^″^ ⊆ *G* ^′^. To see this, suppose for contradiction that $\mu \in [{\kern -2.3pt}[ P^{\prime }_{j} ]{\kern -2.3pt}]_{G^{\prime \prime }}$ for a graph *G* ^″^ as above. Then there must be a child *v* ^′^ of $\mathcal {T}((P^{\prime }_{j})_{\mu _{0}})$ such that *v* ^′^≺ *v*, $\mu _{o}|_{v^{\prime }}\sqsubset \mu $ and *μ*(t r i p l e s(*v* ^′^)) ⊆ *G* ^″^. Since $\mathcal {T}((P^{\prime }_{j})_{\mu _{0}})$ and $\mathcal {T}((P^{\prime }_{j})_{\mu })$ are identical restricted to nodes preceding *v* with respect to ≺, *v* ^′^ is a child of $\mathcal {T}((P^{\prime }_{j})_{\mu })$. Thus, *v* ^′^ is not contained in $\mathcal {T}((P^{\prime }_{j})_{\mu })$, which contradicts maximality of $\mathcal {T}((P^{\prime }_{j})_{\mu })$ since *μ*(t r i p l e s(*v* ^′^)) ⊆ *G* ^″^⊆ *G* ^′^.

If $\mu \notin [{\kern -2.3pt}[ P^{\prime }_{j} ]{\kern -2.3pt}]_{G_{1}}$ for all other *j* as well, then *G*_1_ satisfies all the properties required from *G*. Otherwise we can extend *G*_1_ to a graph *G*_2_ on the base of some other $P^{\prime }_{j}$ with $\mu \in [{\kern -2.3pt}[ P^{\prime }_{j} ]{\kern -2.3pt}]_{G_{1}}$ in the same way as *G*_1_ extends *G*_0_. We then have *G*_2_ ⊆ *G* ^′^, $\mu \in [{\kern -2.3pt}[ P ]{\kern -2.3pt}]_{G_{2}}$, and $\mu \notin [{\kern -2.3pt}[ P^{\prime }_{j} ]{\kern -2.3pt}]_{G_{2}}$ for *j* from both steps. Repeating the extension step until there are no $P^{\prime }_{j}$ having *μ* as a solution on the resulting graph, we obtain a graph that satisfies all the properties required from *G*; in particular, for each *j* the number of added triples to the graph is bounded by $|\mathsf {triples}(P^{\prime }_{j})|$. □

Hardness of equivalence is established in the following lemma by a reduction of ∀∃3SAT, while containment is ${{\Pi }_2^p}$-hard by the results in [35]. Note that both results hold even for fragments without F I L T E R.

### **Lemma 6**

*Problem* Equivalence *(* ${\mathcal {O}_{\text {wwd}}}$ *)* *is* ${{\Pi }_{2}^{p}}$ *-hard* *for the fragment* $\mathcal {O}_{\text {wwd}}$ *of* F I L T E R *-free* *wwd-patterns.*

### *Proof*

We proceed by reduction of the ∀∃3SAT problem, that is, the problem of checking whether a formula of the form

14 $$ \forall \bar{x} \, \exists \bar y \, \psi, $$

holds for a conjunction *ψ* of clauses *t*_1_ ∨ *t*_2_ ∨ *t*_3_ with *t*_*i*_ propositional literals, that is, propositional variables from $\bar {x} \cup \bar y$ or their negations. Without loss of generality, we assume that *ψ* contains no tautologous clauses and no clauses with duplicate literals. Let *ϕ* be a formula of the form (14). Starting from *ϕ*, we construct F I L T E R-free wwd-patterns *P* and *P* ^′^ in O F-normal form, and then show that *ϕ* is true if and only if *P* ≡ *P* ^′^. Let $\bar {x} = x_{1}, \ldots , x_{n}$ and $\bar y = y_{1}, \ldots , y_{m}$.

For each clause *γ* = *t*_1_ ∨ *t*_2_ ∨ *t*_3_, there are exactly 7 assignments to the variables in *t*_1_, *t*_2_, *t*_3_ making *γ* true, and exactly one assignment making *γ* false (since *γ* is assumed to be non-tautologous and contain no duplicate literals). Let, for each such *γ* in *ψ*, each *ℓ*, 1 ≤ *ℓ* ≤ 7, and each *j*, 1 ≤ *j* ≤ 3, *v* *a* *l*(*γ*, *j*, *ℓ*) = ⊤ if the variable of literal *t*_*j*_ evaluates to true in the *ℓ*’th assignment making *γ* true, and *v* *a* *l*(*γ*, *j*, *ℓ*) = ⊥, otherwise; here ⊤ and ⊥ are fresh IRIs. Let also, for every clause *γ* in *ψ*, $\mathit {cl}_{\gamma }^{1} ,\dots ,\mathit {cl}_{\gamma }^{7} $ and $\mathit {lit}_{\gamma }^{1} $, $\mathit {lit}_{\gamma }^{2} $, $\mathit {lit}_{\gamma }^{3} $ be fresh IRIs. We define, for each *γ* and 1 ≤ *ℓ* ≤ 7, a basic pattern

$$B^{\ell}_{\gamma} = \{{(\mathit{cl}_{\gamma}^{\ell}, \mathit{lit}_{\gamma}^{1} , \mathit{val}(\gamma,1,\ell) )}, {(\mathit{cl}_{\gamma}^{\ell}, \mathit{lit}_{\gamma}^{2} , \mathit{val}(\gamma,2,\ell) )}, {(\mathit{cl}_{\gamma}^{\ell} , \mathit{lit}_{\gamma}^{3} , \mathit{val}(\gamma,3,\ell) )}\}, $$

and a basic pattern

$$B^{*}_{\gamma} = B^{1}_{\gamma} \cup {\dots} \cup B^{7}_{\gamma} $$

(note that these patterns do not have any variables).

Let, for each propositional variable $z \in \bar {x} \cup \bar y$, *i* *r* *i*_*z*_ be a fresh IRI and ?*z* be a fresh SPARQL variable. For each *γ*, let also ?*c*_*γ*_, $?v_{\gamma }^{1} $, $?v_{\gamma }^{2} $, $?v_{\gamma }^{3} $ be fresh variables. Let

$$\begin{array}{@{}rcl@{}} B_{\gamma} &=& \{{(?c_{\gamma} , \mathit{lit}_{\gamma}^{1} , ?v_{\gamma}^{1} )}, {(?c_{\gamma} , \mathit{lit}_{\gamma}^{2} , ?v_{\gamma}^{2} )}, {(?c_{\gamma}, \mathit{lit}_{\gamma}^{3} , ?v_{\gamma}^{3} )},\\ &&{(\mathit{var}_{\gamma}^{1} , \mathit{iri}_{\gamma}^{1} , ?v_{\gamma}^{1} )}, {(\mathit{var}_{\gamma}^{2} , \mathit{iri}_{\gamma}^{2} , ?v_{\gamma}^{2} )}, {(\mathit{var}_{\gamma}^{3} , \mathit{iri}_{\gamma}^{3} , ?v_{\gamma}^{3} )}\}, \end{array} $$

where each $\mathit {var}_{\gamma }^{j} $ and $\mathit {iri}_{\gamma }^{j} $, 1 ≤ *j* ≤ 3, are the variable and the IRI corresponding to the variable of literal *t*_*j*_ in *γ*; that is, $\mathit {var}_{\gamma }^{j} ={?z}$ and $\mathit {iri}_{\gamma }^{j} = iri_{z}$ if *t*_*j*_ = *z* or *t*_*j*_ = ¬*z*.

Let ?*u* and ?*s* be fresh variables and *r*, *c* *y* ^⊥^, *c* *y* ^⊤^ fresh IRIs. We define

$$\begin{array}{lcll} B_{\text{base}} & = & \{{(?u, iri_{x_{1}}, \top)}, \dots, {(?u, iri_{x_{n}}, \top)}\}, \\ B_{i}^{\bot} & = & \{{({?x_{i}}, {iri_{x_{i}}}, {\bot})}\}, & \text{ for all } 1 \leq i \leq n, \\ B_{i}^{\top} & = & \{{({?x_{i}}, {iri_{x_{i}}}, {\top})}\}, & \text{ for all } 1 \leq i \leq n, \\ B_{\text{valid}} & = & \{{({?s}, {r}, {?s})}, \\ && ~\,{(cy^{\bot}, iri_{y_{1}}, \bot)}, \dots, {(cy^{\bot}, iri_{y_{m}}, \bot)}, {} \\ && ~\,{(cy^{\top}, iri_{y_{1}}, \top)}, \dots, {(cy^{\top}, iri_{y_{m}}, \top)}\} \cup {} \\ && B^{*}_{\gamma_{1}} \cup {\dots} \cup B^{*}_{\gamma_{k}}, & \text{ where } \psi = \gamma_{1} \wedge {\dots} \wedge \gamma_{k}, \\ B_{\psi} & = & \{{(?s, r, ?s)}\} \cup B_{\gamma_{1}} \cup {\dots} \cup B_{\gamma_{k}}, & \text{ where } \psi = \gamma_{1} \wedge {\dots} \wedge \gamma_{k}. \end{array} $$

For example, a visualisation of these patterns for

$$\phi=\forall x_{1}, x_{2} \ \exists y_{1}, y_{2} \ (\neg x_{1}\lor y_{1}\lor y_{2})\land(\neg y_{1}\lor\neg y_{2}\lor x_{2}) $$

is shown in Fig. 6.

**Fig. 6:**
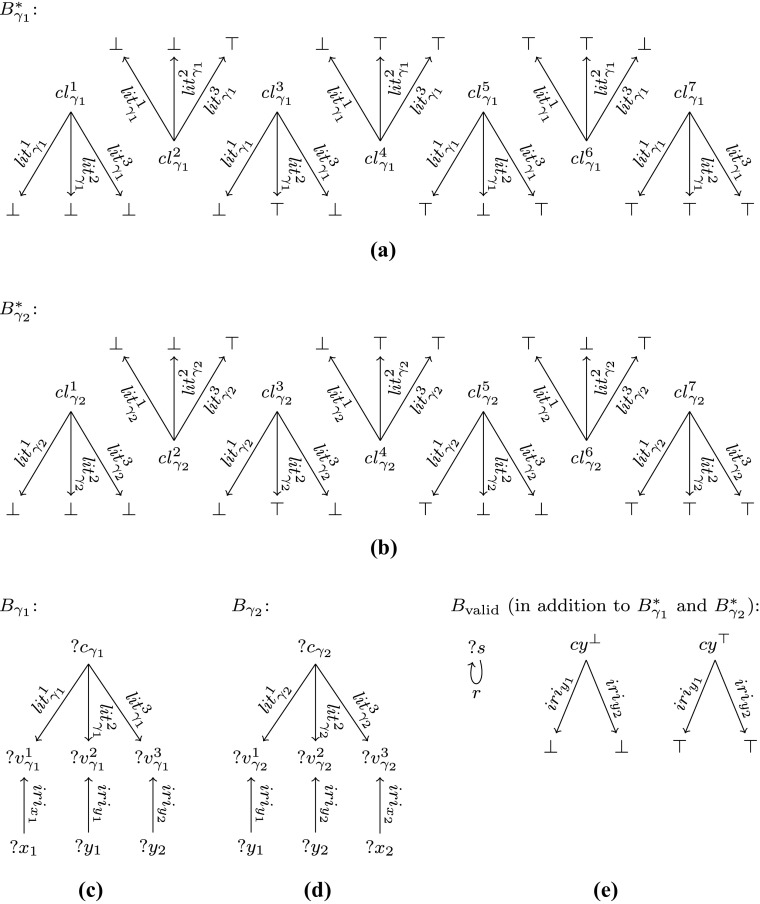
Visualisation of the patterns **a** $B_{\gamma _{1}}^{*}$, **b** $B_{\gamma _{2}}^{*}$, **c** $B_{\gamma _{1}}$, **d** $B_{\gamma _{2}}$, and **e** *B*_valid_ used in the proof of Lemma 6 on the example formula ∀*x*_1_, *x*_2_ ∃*y*_1_, *y*_2_ *γ*_1_ ∧ *γ*_2_ with *γ*_1_ = ¬*x*_1_ ∨ *y*_1_ ∨ *y*_2_ and *γ*_2_ = ¬*y*_1_ ∨ ¬*y*_2_ ∨ *x*_2_

Finally, let

$$P = (({\dots} ((B_{\text{base}}~{{\mathsf{OPT}}}~B_{1}^{\bot})~{{\mathsf{OPT}}}~B_{1}^{\top})~{{\mathsf{OPT}}}~ \ldots~{{\mathsf{OPT}}}~B_{n}^{\bot})~{{\mathsf{OPT}}}~B_{n}^{\top})~{{\mathsf{OPT}}}~B_{\psi}, $$

and

$$\begin{array}{@{}rcl@{}} P^{\prime} = ((({\dots} ((B_{\text{base}}~{{\mathsf{OPT}}}~B_{1}^{\bot})~{{\mathsf{OPT}}}~B_{1}^{\top})~{{\mathsf{OPT}}}~\ldots~{{\mathsf{OPT}}}\! &\,B_{n}^{\bot})&{{\mathsf{OPT}}}~B_{n}^{\top})\\ && {{\mathsf{OPT}}}~B_{\text{valid}})~{{\mathsf{OPT}}}~B_{\psi} \end{array} $$

be two F I L T E R-free wwd-patterns in O F-normal form.

We next show that *ϕ* is true if and only if *P* is equivalent to *P* ^′^, starting with the forward direction.

Let *ϕ* be true, yet, for the sake of contradiction, *P* is not equivalent to *P* ^′^. Then there is a graph *G* and mapping *μ* such that *μ* ∈ [[*P*]]_*G*_, but *μ* ∉ [[*P* ^′^]]_*G*_. Since patterns *P* and *P* ^′^ have the same root *B*_base_, which contains ?*u* as the only variable, we conclude that ?*u* ∈ *d* *o* *m*(*μ*). Each ?*x*_*i*_ is also in *d* *o* *m*(*μ*) by the construction of *P*, since there is a homomorphism from the corresponding leaf $B_{i}^{\top }$ to the root *B*_base_. However, it is not necessary that ${(\mu (?x_{i}), iri_{x_{i}}, \top )}$ is in *G* because if *G* contains a triple of the form ${(c, iri_{x_{i}}, \bot )}$ for some IRI *c*, we will have ${(\mu (?x_{i}), iri_{x_{i}}, \bot )}\in G$. Note also that nothing prevents *G* from containing both a triple ${(c, iri_{x_{i}}, \bot )}$ and a triple ${(c, iri_{x_{i}}, \top )}$ for some *i*. Depending on whether ?*s* ∈ *d* *o* *m*(*μ*) or not, we have two cases.

### **Case 1**

Let ?*s* ∈ *d* *o* *m*(*μ*), that is, there is a homomorphism from *B*_*ψ*_ to *G* that aligns with the previous assignment of all ?*x*_*i*_. In particular, this means that *d* *o* *m*(*μ*) = *v* *a* *r* *s*(*P*) = *v* *a* *r* *s*(*P* ^′^). If there is no homomorphism from *B*_valid_ to *G*, then *μ* ∈ [[*P* ^′^]]_*G*_, because *B*_*ψ*_ is the last leaf of *P* ^′^ as well, and nothing prevents it from matching. But this contradicts the assumption. However, even if there is a homomorphism h from *B*_valid_ to *G*, we still have a contradiction because $\mu \in [{\kern -2.3pt}[ P^{\prime } ]{\kern -2.3pt}]_{G}$ still holds. Indeed, ?*s* is the only variable in *B*_valid_ and is essentially isolated in *B*_valid_, so if *h* is a homomorphism from *B*_valid_ to *G*, then *h* ^′^, which maps ?*s* to *μ*(?*s*), is also such a homomorphism (in other words, since by the assumptions of this case, we know that (?*s*, *r*, ?*s*) has a match in *G*, the existence of *h* just means that all the ground triples of *B*_valid_ are in *G*). This means, however, that nothing prevents *B*_*ψ*_ from matching in *P* ^′^, implying *μ* ∈ [[*P* ^′^]]_*G*_.

### **Case 2**

Let ?*s* ∉ *d* *o* *m*(*μ*). Since *μ* ∉ [[*P* ^′^]]_*G*_, there is no homomorphism from *B*_*ψ*_ to *G* but there is one from *B*_valid_ to *G*, that is, all ground triples of *B*_valid_ are in *G* (the non-existence of a homomorphism from *B*_*ψ*_ to *G* is immediate since ?*s* ∉ *d* *o* *m*(*μ*) and *μ* ∈ [[*P*]]_*G*_; the existence of a homomorphism from *B*_valid_ to *G* then follows since otherwise we would have $\mu \in [{\kern -2.3pt}[ P^{\prime } ]{\kern -2.3pt}]_{G}$). Consider now a truth assignment *α* of variables $\bar {x}$ such that if *α*(*x*_*i*_) is true then ${(\mu (?x_{i}), iri_{x_{i}}, \top )} \in G$ and if *α*(*x*_*i*_) is false then ${(\mu (?x_{i}), iri_{x_{i}}, \bot )} \in G$ (as we mentioned earlier, *α* may be not unique, but the argument does not depend on its uniqueness). Since *ϕ* is true, we know that *α* can be extended to the variables $\bar y$ in such a way that each clause in *ψ* holds. Let *α* ^′^ be such an extension, and let *μ* ^′^ be an extension of *μ* to the variables in *B*_*ψ*_ such that, for all *j*, *μ* ^′^(?*y*_*j*_) = *c* *y* ^⊤^ if *α* ^′^(*y*_*j*_) is true and *μ* ^′^(?*y*_*j*_) = *c* *y* ^⊥^ otherwise. Then, for every clause *γ* in *ψ*, the IRIs $\mu ^{\prime }(?v_{\gamma }^{1} )$, $\mu ^{\prime }(?v_{\gamma }^{2})$, $\mu ^{\prime }(?v_{\gamma }^{3} )$ correspond to the values *α* ^′^(*z*_1_), *α* ^′^(*z*_2_), *α* ^′^(*z*_3_), respectively, where *z*_1_, *z*_2_, *z*_3_ are the variables in the literals *t*_1_, *t*_2_, *t*_3_ of *γ*. Moreover, $\mu ^{\prime }(?c_{\gamma }) = \mathit {cl}_{\gamma }^{\ell } $ for *ℓ* the number of the assignment *α* ^′^(*z*_1_), *α* ^′^(*z*_2_), *α* ^′^(*z*_3_); this assignment makes *γ* true by the choice of *α* ^′^ (in other words, for every *γ* there is some *ℓ* such that ${(\mu ^{\prime }(?c_{\gamma }), \mathit {lit}_{\gamma }^{i}, \mu ^{\prime }(?v_{\gamma }^{i} ))}\in B_{\gamma }^{\ell }$ for all 1 ≤ *i* ≤ 3). Hence, the extension *μ* ^′^ is contained in [[*P*]]_*G*_, and hence *μ* ∉ [[*P*]]_*G*_, which, however, contradicts the original assumption.

Since both cases yield a contradiction, we conclude that *P* is equivalent to *P* ^′^.

We continue with the backward direction of the equivalence. Suppose that *P* ≡ *P* ^′^, yet, for the sake of contradiction, *ϕ* is false. Then, there is a truth assignment *α* of the variables $\bar {x}$ such that for each extension *α* ^′^ of *α* to the variables $\bar y$ there is a clause *γ* in *ψ* that evaluates to false under *α* ^′^. Fix such an *α* and consider the graph *G* consisting of

- the triple ${(u, iri_{x_{i}}, \top )}$, for each *x*_*i*_ in $\bar {x}$ and a fresh IRI *u*;
- the triple ${(c_{x_{i}}, iri_{x_{i}}, \top )}$, for each *x*_*i*_ in $\bar {x}$ with *α*(*x*_*i*_) true, and the triple ${({c_{x_{i}}}, {iri_{x_{i}}}, {\bot })}$, for each *x*_*i*_ in $\bar {x}$ with *α*(*x*_*i*_) false, where $c_{x_{1}},\dots ,c_{x_{n}}$ are fresh IRIs;
- all ground triples from *B*_valid_, that is, all of its triples except (?*s*, *r*, ?*s*);
- the triple (*s*, *r*, *s*) for a fresh IRI *s*.

Consider also the mapping *μ* such that

- *μ*(?*u*) = *u*;
- $\mu (?x_{i}) = c_{x_{i}}$, for each *x*_*i*_ in $\bar {x}$.

Clearly, *μ* is a pp-solution to both *P* and *P* ^′^ over *G*. However, by the construction of *G*, the mapping *μ* ^′^ = *μ* ∪ {?*s* ↦ *s*} is a pp-solution to *P* ^′^ over *G* as well. Thus, *μ* is not a solution to *P* ^′^ over *G*, and, since *P* ⊆ *P* ^′^, we also have *μ* ∉ [[*P*]]_*G*_. Consequently, it must be possible to further extend *μ* ^′^ to a mapping *μ* ^″^ that is both in [[*P*]]_*G*_ and in [[*P* ^′^]]_*G*_, and is defined on all variables in *B*_*ψ*_. Essentially, this means that there is a homomorphism from *B*_*ψ*_ to *B*_valid_ that preserves ?*s*. Consider now the extension *α* ^′^ of *α* to the variables $\bar y$ such that *μ* ^″^(?*y*_*j*_) = *c* *y* ^⊤^ if *α* ^′^(*y*_*j*_) is true and *μ* ^″^(?*y*_*j*_) = *c* *y* ^⊥^ otherwise. By the construction of *B*_valid_, this assignment validates all clauses in *ψ*, which, however, contradicts the assumption that *ϕ* is false.

Thus, we have shown that *ϕ* is true if and only if *P* ≡ *P* ^′^. □

### **Theorem 7**

*Problems* $\textsc {Equivalence}({\mathcal {L}})$ *and* $\textsc {Containment}({\mathcal {L}})$ *are both* ${{\Pi }_2^p}$ *-complete* *for any* $\mathcal {L}\!\in \!\{\mathcal {P}_{\text {wwd}}, \mathcal {U}_{\text {wwd}}\}$ *.*

### *Proof*

The existence of a ${{\Pi }_2^p}$ algorithm for containment immediately follows from Lemma 5: to show that *P* ⊈ *P* ^′^, for $P,P^{\prime }\in \mathcal {P}_{\text {wwd}}$, we just need to guess, in NP, a graph *G* of linear size as well as a mapping *μ*, check that *μ* ∉ [[*P* ^′^]]_*G*_, and then call for a coNP oracle for checking that *μ* ∈ [[*P*]]_*G*_. The claim for patterns in $\mathcal {U}_{\text {wwd}}$ is similar, but involves guessing a disjunct *P*_1_ of *P* with *μ* ∈ [[*P*_1_]]_*G*_ and checking $\mu \notin [{\kern -2.3pt}[ {P^{\prime }_{1}} ]{\kern -2.3pt}]_{G} $ for every disjunct $P^{\prime }_{1}$ of *P* ^′^. Since *P* ≡ *P* ^′^ if and only if containment holds in both directions, the problem $\textsc {Equivalence}({\mathcal {U}_{\text {wwd}}})$ is also in ${{\Pi }_{2}^{p}}$.

Hardness follows by the results in [35] for containment and by Lemma 6 for equivalence. □

Hence, for U N I O N- and F I L T E R-free patterns, the step from well-designed to weakly well-designed O P T incurs a complexity jump for containment and equivalence. However, for the fragments with U N I O N or projection complexity remains the same in both cases. As far as we are aware, these are the first decidability results on query equivalence and related problems for SPARQL fragments with O P T and F I L T E R.

## Analysis of DBpedia Logs

In this section, we present an analysis of query logs over DBpedia, which suggests that the step from wd-patterns to wwd-patterns makes a dramatic difference in real life: while only about half of the queries with O P T have well-designed patterns, almost all of these patterns fall into the weakly well-designed fragment.

DBpedia [26] is a project providing access to RDF data extracted from Wikipedia via a SPARQL endpoint. DBpedia query logs are well suited for analysing the structure of real-life SPARQL queries as they contain a large amount of general-purpose knowledge base queries, generated both manually and automatically. DBpedia query logs have been analysed by Picalausa and Vansummeren [34], who reported that, over a period in 2010, about 46.38% of a total of 1344K distinct DBpedia queries used O P T. However, only 47.80% of the queries with O P T had well-designed patterns. Another analysis of DBpedia logs from the USEWOD 2011 data set performed by Arias Gallego et al. [7] concluded that 16.61% of about 5166K queries contained O P T; however, detailed structure of queries was not analysed.

We considered query logs over DBpedia 3.9 from USEWOD 2015 [30] and USEWOD 2016 [29]. The USEWOD 2015 DBpedia dataset is a random selection of almost 14M queries from the first half of 2014 while the USEWOD 2016 dataset contains 35M queries from the second half of 2015. We removed syntactically incorrect queries as well as queries outside of $\mathcal {S}$ (in particular, queries using operators specific to SPARQL 1.1). Also, we rewrote the patterns of the remaining queries to unions of U N I O N-free patterns as proposed in [33] and eliminated duplicates, which left us with 6.6M queries in USEWOD 2015 and 9.1M queries in USEWOD 2016. Finally, we isolated queries involving O P T and counted how many of their patterns were in $\mathcal {U}_{\text {wd}}$ and in $\mathcal {U}_{\text {wwd}}$.

The results are given in Table 1. They confirm that a non-negligible number of DBpedia queries use O P T (over 17%). However, by far not all queries with O P T are well-designed (only about 44% for USEWOD 2015 and 52% for USEWOD 2016), which is consistent with the results in [34]. On the other hand, almost all of the patterns with O P T (over 99.9% in both cases) are weakly well-designed, which we consider as the main practical justification for wwd-patterns.

**Table 1:** Structure of query patterns in DBpedia logs from USEWOD 2015 and 2016

	USEWOD 2015			USEWOD 2016		
	Unique patterns	Fraction of total	Fraction of patterns with *O* *P* *T*	Unique patterns	Fraction of total	Fraction of patterns with *O* *P* *T*
Total	6 606 201	100%		9 119 492	100%	
Patterns with *O* *P* *T*	1 147 704	17.37%	100%	1 582 698	17.36%	100%
Unions of wd-patterns	500 676	7.58%	43.62%	816 276	8.95%	51.57%
Unions of wwd-patterns	1 147 135	17.36%	99.95%	1 582 339	17.35%	99.98%

What about the remaining 0.05% of queries with O P T? We looked at a number of such queries and identified what we believe to be the three most common sources of non-weakly-well-designedness in query patterns. The first and seemingly most common such source is joins between an O P T subpattern and another pattern on a variable that only occurs in the right argument of the O P T subpattern. The following query is an example of such a join:

15 $$\begin{array}{@{}rcl@{}} \mathsf{SELECT} &?\ell,?u& \mathsf{WHERE} \; \\ && ({(?s, \texttt{label}, ?\ell)}~{{\mathsf{OPT}}}~{(?s, \texttt{type}, ?t)})~{{\mathsf{AND}}}~{(?t, \texttt{subClassOf}, ?u)}.\\ \end{array} $$

**Fig. 7:**
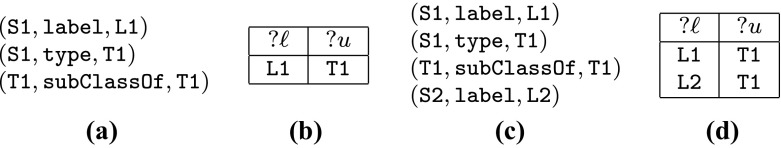
**a** Graph *G*; **b** answers to query (15) over *G*; **c** graph *G* ^′^; **d** answers over *G* ^′^

We believe that the vast majority if not all such queries are erroneous as they are highly unlikely to yield meaningful answers in case the optional part fails to match. Intuitively, one would expect an answer to query (15) to contain the label of an object in variable ?*ℓ* together with one of its supertypes in variable ?*u*. And indeed, this is the answer returned by the query on graph *G* in Fig. 7a (see Fig. 7b). However, if an object in ?*s* is not assigned any type, which is explicitly allowed by the use of O P T, the query does not just return its label in ?*ℓ* leaving ?*u* unbound, as one would expect; instead, it returns the cross product of the label and all types in the graph, which are, of course, completely unrelated to the object in ?*s* (see Fig. 7c and d).

A second source of non-weakly-well-designed patterns is joins between an O P T subpattern and another pattern where the left argument of the O P T subpattern is empty. The following query illustrates this source:

16 $$\begin{array}{@{}rcl@{}} \mathsf{SELECT} &?b,?h,?c& \mathsf{WHERE} \; \\ && {(?b, \texttt{height}, ?h)}~{{\mathsf{AND}}}~(\emptyset~{{\mathsf{OPT}}}~{(?b, \texttt{city}, ?c)}).\\ \end{array} $$

Intuitively, query (16) computes the join between the answers to the pattern (?*b*,height, ?*h*) and those to (?*b*,city, ?*c*), provided the second set is non-empty; otherwise, the query just returns the answers to (?*b*,height, ?*h*). Hence, query (16) is equivalent to the following query in $\mathcal {S}_{\text {wwd}}$:

$$\begin{array}{@{}rcl@{}} \mathsf{SELECT} &?b,?h,?c& \mathsf{WHERE} \; \\ &&(({(?b, \texttt{height}, ?h)}~{{\mathsf{OPT}}}~{(?b^{\prime}, \texttt{city}, ?c^{\prime})})~{{\mathsf{FILTER}}}\neg\mathit{bound}(?c^{\prime})) \\&&{{\mathsf{UNION}}}~({(?b, \texttt{height}, ?h)}~{{\mathsf{AND}}}~{(?b, \texttt{city}, ?c)}). \end{array} $$

We conclude that, while queries such as (16) may make sense in practice, they can be easily and intuitively restated using wwd-patterns.

Our final, and most interesting, source of real-life non-wwd-patterns is U N I O N in the right argument of O P T. For instance, consider the pattern

17 $$\begin{array}{*{20}l} {(?p, \texttt{type}, \texttt{person})}~{{\mathsf{OPT}}}~({(?p, \texttt{son}, ?a)}~{{\mathsf{UNION}}}~{(?p, \texttt{daughter}, ?a)}). \end{array} $$

The pattern is quite intuitive and it is easy to imagine similar patterns being useful in various applications. However, the normalisation algorithm in [33], which “pushes” unions outside, converts (17) to a pattern that is inherently non-well-designed (due to the two occurrences of ?*a* in the third disjunct):

$$\begin{array}{@{}rcl@{}} &&({(?p, \texttt{type}, \texttt{person})}~{{\mathsf{AND}}}~{(?p, \texttt{son}, ?a)})\\ &&~~~{{\mathsf{UNION}}}~\\ &&({(?p, \texttt{type}, \texttt{person})}~{{\mathsf{AND}}}~{(?p, \texttt{daughter}, ?a)})\\ &&~~~{{\mathsf{UNION}}}~\\ &&((({(?p, \texttt{type}, \texttt{person})}~{{\mathsf{OPT}}}~({(?p, \texttt{son}, ?a)}~{{\mathsf{AND}}}~{(?x_{1}, ?y_{1}, ?z_{1})}))~{{\mathsf{AND}}} {} \\ &&~\,\,\,({(?p, \texttt{type}, \texttt{person})}~{{\mathsf{OPT}}}~({(?p, \texttt{daughter}, ?a)}~{{\mathsf{AND}}}~{(?x_{2}, ?y_{2}, ?z_{2})})))\\ &&\qquad\!{{\mathsf{FILTER}}}~\neg\mathit{bound}(?x_{1})\land\neg\mathit{bound}(?x_{2})). \end{array} $$

We believe that this behaviour is unavoidable in general as we expect query answering to become ${{\Pi }_2^p}$-hard over patterns that contain U N I O N in the right argument of O P T. Yet, for certain classes of patterns, generalising (17), one may be able to obtain a coNP evaluation algorithm by accounting for U N I O N natively rather than relying on the normalisation in [33]. A detailed study of such patterns, however, is outside the scope of the present paper.

## Conclusion and Future Work

In this paper, we introduced a new fragment of SPARQL patterns called weakly well-designed patterns. This fragment extends the widely studied well-designed fragment by allowing variables from the optional side of an O P T-subpattern that are not “guarded” by the mandatory side to occur in certain positions outside of the subpattern. We showed that queries with wwd-patterns enjoy the same low complexity of evaluation as well-designed queries but cover almost all real-life queries. Moreover, our fragment is the maximal coNP fragment that does not impose structural restrictions on basic patterns and filter conditions. We studied the expressive power of the fragment and the complexity of its query optimisation problems.

For future work, we want to extend wwd-patterns to allow for non-top-level occurrences of U N I O N and projection. As we have seen in the previous section, this promises to be a challenging task since a naive extension of our definitions to such constructs is likely to increase reasoning complexity. Also, we want to take into account features of SPARQL 1.1 [17] such as G R A P H, N O T E X I S T S and property paths. Finally, we would like to implement our ideas in a prototype system and compare its performance with existing SPARQL engines.
